# Supplementary material for: Interventions to improve perinatal outcomes among migrant women in high-income countries: a systematic review protocol
Source: BMJ Open. 2023 Aug 17;13(8):e072090. doi: 10.1136/bmjopen-2023-072090 (PMC10441090; doi:10.1136/bmjopen-2023-072090)
Supplement: Supplementary data [file bmjopen-2023-072090supp001.pdf]

APPENDIX 1 – Search Strategies

Emcare

1 (Aruba* or Andorra* or "United Arab Emirates" or UAE or Emirati* or Antigua* or Barbuda* or Australia* or Austria* or Belgi* or Bahrain* or Baham* or Bermuda* or Barbad* or Brunei* or Canada* or Switzerland or Swiss or "Channel Island*" or Chile or Chilean* or Curacao* or "Cayman Island*" or Caymanian* or Cypr* or Czech* or German* or Denmark or Danish or Spain* or Estonia* or Finland or Finnish or France or French or Faroe* or "United Kingdom" or UK or England or Wales or welsh or Scotland or Brit* or Scot* or English or Gibraltar * or Gree* or Greenland* or Guam* or Chamorros or Hong Kong* or Croatia* or Hungar* or "Isle of Man" or Manx or Ireland or Irish or Iceland* or Israel* or Ital* or Japan* or "St Kitts" or Korea* or Kuwait* or Liechtenstein* or Lithuania* or Luxembourg* or Latvia* or Maca* or "St Martin*" or "Sint Maarten*" or Monaco or Monegasqu* or Malta or Maltese or "Northern Mariana Island*" or "New Caledonia*" or Netherlands or Dutch or Norw* or Nauru* or "New Zealand" or Kiwi or Oman* or Palau* or Poland or Polish or "Puerto Ric*" or Portug* or "French Polynesia*" or Qatar* or "Saudi Arabia*" or Singapor* or "San Marino" or "Sammarinese" or Slovak* or Slovenia* or Sweden or Swedish or Seychell* or Creole or "Turks Caicos" or Trinidad* or Tobago* or Uruguay* or "United States" or USA or America* or Venezuela* or "Virgin Island*" or EU or "European Union" or European*).ti,ab. 1458038

2 developed country/ or european union/ 13481

3 developed countr*.mp. 13352

4 (high income countr* or high-income countr*).mp. 6622

5 industrialised countr*.mp. 623

6 more economically developed countr*.mp. 22

7 MEDC.mp. 8

8 advanced countr*.mp. 158

9 advanced econom*.mp. 92

10 developed econom*.mp. 161

11 industrialized econom*.mp. 11

12 industrialised econom*.mp. 8

13 (Migrant* or refugee* or immigrant* or foreigner* or newcomer* or new-comer* or migration or immigration or emigrant* or nonnative* or non-native* or diaspora or border crossing* or transient* or asylum-seek*).mp. 163314

14 ((displaced or undocumented or foreign* or resettle* or settle* or displace*) adj3 (person* or people)).mp. 1301

15 exp Migration/ 8976

16 exp Refugee/ 7217

17 13 or 14 or 15 or 16 163953

18 (pregnan* or postpartum or post-partum or postnatal or post-natal or puerper* or antenatal or ante-natal or prenatal or pre-natal or antepartum or ante-partum or peripartum or peri-partum or birth* or trimester* or mother* or mum or mums or mom or moms or perinatal or peri-natal or childbirth* or obstetric* or maternity or maternal).mp. 460639

19 exp perinatal period/ or exp puerperium/ or exp pregnancy/ 125861

20 18 or 19 464076

21 (intervention* or prevent* or program* or strateg* or class* or special*).mp. 2320749

22 (Peer-led or "peer led" or doula* or midwi*).mp. 22932

23 ((antenatal or Postnatal) and (session* or lesson* or workshop*)).mp. 1060

24 21 or 22 or 23 2333617

25 1 and 17 and 20 and 24 2274

26 2 or 3 or 4 or 5 or 6 or 7 or 8 or 9 or 10 or 11 or 12 27873

27 17 and 20 and 26 212

28 25 or 27 2410

Embase

1 (Aruba* or Andorra* or "United Arab Emirates" or UAE or Emirati* or Antigua* or Barbuda* or Australia* or Austria* or Belgi* or Bahrain* or Baham* or Bermuda* or Barbad* or Brunei* or Canada* or Switzerland or Swiss or "Channel Island*" or Chile or Chilean* or Curacao* or "Cayman Island*" or Caymanian* or Cypr* or Czech* or German* or Denmark or Danish or Spain* or Estonia* or Finland or Finnish or France or French or Faroe* or "United Kingdom" or UK or England or Wales or welsh or Scotland or Brit* or Scot* or English or Gibraltar * or Gree* or Greenland* or Guam* or Chamorros or Hong Kong* or Croatia* or Hungar* or "Isle of Man" or Manx or Ireland or Irish or Iceland* or Israel* or Ital* or Japan* or "St Kitts" or Korea* or Kuwait* or Liechtenstein* or Lithuania* or Luxembourg* or Latvia* or Maca* or "St Martin*" or "Sint Maarten*" or Monaco or Monegasqu* or Malta or Maltese or "Northern Mariana Island*" or "New Caledonia*" or Netherlands or Dutch or Norw* or Nauru* or "New Zealand" or Kiwi or Oman* or Palau* or Poland or Polish or "Puerto Ric*" or Portug* or "French Polynesia*" or Qatar* or "Saudi Arabia*" or Singapor* or "San Marino" or "Sammarinese" or Slovak* or Slovenia* or Sweden or Swedish or Seychell* or Creole or "Turks Caicos" or Trinidad* or Tobago* or Uruguay* or "United States" or USA or America* or Venezuela* or "Virgin Island*" or EU or "European Union" or European*).ti,ab. 5387298

2 developed country/ or european union/ 64842

3 developed countr*.mp. 65772

4 (high income countr* or high-income countr*).mp. 16400

5 industrialised countr*.mp. 1957

6 more economically developed countr*.mp. 49

7 MEDC.mp. 51

8 advanced countr*.mp. 668

9 advanced econom*.mp. 218

10 developed econom*.mp. 516

11 industrialized econom*.mp. 40

12 industrialised econom*.mp. 11

13 (Migrant* or refugee* or immigrant* or foreigner* or newcomer* or new-comer* or migration or immigration or emigrant* or nonnative* or non-native* or diaspora or border crossing* or transient* or asylum-seek*).mp. 1056344

14 ((displaced or undocumented or foreign* or resettle* or settle* or displace*) adj3 (person* or people)).mp. 2841

15 exp Migration/ 48713

16 exp Refugee/ 16436

17 13 or 14 or 15 or 16 1057673

18 (pregnan* or postpartum or post-partum or postnatal or post-natal or puerper* or antenatal or ante-natal or prenatal or pre-natal or antepartum or ante-partum or peripartum or peri-partum or birth* or trimester* or mother* or mum or mums or mom or moms or perinatal or peri-natal or childbirth* or obstetric* or maternity or maternal).mp. 2417880

19 exp perinatal period/ or exp puerperium/ or exp pregnancy/ 824664

20 18 or 19 2436673

21 (intervention* or prevent* or program* or strateg* or class* or special*).mp. 9841412

22 (Peer-led or "peer led" or doula* or midwi*).mp. 47440

23 ((antenatal or Postnatal) and (session* or lesson* or workshop*)).mp. 3201

24 21 or 22 or 23 9869943

25 1 and 17 and 20 and 24 6620

26 2 or 3 or 4 or 5 or 6 or 7 or 8 or 9 or 10 or 11 or 12 114481

27 17 and 20 and 26 956

28 25 or 27 7319

SCOPUS

TITLE-ABS-KEY (aruba* OR andorra* OR "United Arab Emirates" OR uae OR emirati* OR antigua* OR barbuda* OR australia* OR austria* OR belgi* OR bahrain* OR baham* OR bermuda* OR barbad* OR brunei* OR canada* OR switzerland OR swiss OR "Channel Islands" OR chile OR chilean* OR curacao* OR "Cayman Islands" OR caymanian* OR cypr* OR czech* OR german* OR denmark OR danish OR spain* OR estonia* OR finland OR finish OR france OR french OR faroe* OR

"United Kingdom" OR uk OR england OR wales OR scotland OR brit* OR scot* OR english OR "Gibraltar *" OR gree* OR greenland* OR guam* OR chamorros OR "Hong Kong*" OR croatia* OR hungar* OR "Isle of Man" OR manx OR ireland OR irish OR iceland* OR israel* OR ital* OR japan* OR "St Kitts" OR korea* OR kuwait* OR liechtenstein* OR lithuania* OR luxembourg* OR latvia* OR maca* OR "St Martin*" OR "Sint Maarten*" OR monaco OR monegasqu* OR malta OR maltese OR "Northern Mariana Island*" OR "New Caledonia*" OR netherlands OR dutch OR norw* OR nauru* OR "New Zealand" OR kiwi OR oman* OR palau* OR poland OR polish OR "Puerto Ric*" OR portug* OR "French Polynesia*" OR qatar* OR "Saudi Arabia*" OR singapor* OR "San Marino" OR sammarinese OR slovak* OR slovenia* OR sweden OR swedish OR seychell* OR creole OR "Turks Caicos" OR trinidad* OR tobago* OR uruguay* OR "United States" OR usa OR america* OR venezuela* OR "Virgin Island*" OR eu OR "European Union" OR european* OR "high income countr*" OR "high-income countr*" OR "industrialised countr*" OR "more economically developed countr*" OR "advanced countr*" OR "advanced econom*" OR "MEDC" OR "developed econom*" OR "industrialized econom*" OR "industrialised econom*") **AND** TITLE-ABS-KEY (migrant* OR refugee* OR immigrant* OR foreigner* OR newcomer* OR new-comer* OR migration OR immigration OR emigrant* OR nonnative* OR non-native* OR diaspora OR "border crossing*" OR transient* OR asylum-seek* OR "Displaced people*" OR "undocumented people*" OR "foreign* people*" OR "resettle* people*" OR "settle* people*" OR "Displaced person*" OR "undocumented person*" OR "foreign* person*" OR "resettle* person*" OR "settle* person*") **AND** TITLE-ABS-KEY (pregnan* OR postpartum OR post-partum OR postnatal OR post-natal OR puerperal OR antenatal OR ante-natal OR prenatal OR pre-natal OR antepartum OR ante-partum OR peripartum OR peri-partum OR birth* OR trimester* OR mother* OR mum OR mums OR mom OR moms OR perinatal OR peri-natal OR childbirth* OR obstetric* OR puerper* OR maternity OR maternal) **AND** TITLE-ABS-KEY ((intervention* OR prevent* OR program* OR strateg* OR class* OR special* OR peer-led OR "peer led" OR doula* OR midwi*) OR ((antenatal OR postnatal) AND (class* OR session* OR lesson* OR workshop*)))

8815

MEDLINE

Ovid MEDLINE(R) ALL <1946 to December 09, 2022>

1 (Aruba* or Andorra* or "United Arab Emirates" or UAE or Emirati* or Antigua* or Barbuda* or Australia* or Austria* or Belgi* or Bahrain* or Baham* or Bermuda* or Barbad* or Brunei* or Canada* or Switzerland or Swiss or "Channel Island*" or Chile or Chilean* or Curacao* or "Cayman Island*" or Caymanian* or Cypr* or Czech* or German* or Denmark or Danish or Spain* or Estonia* or Finland or Finnish or France or French or Faroe* or "United Kingdom" or UK or England or Wales or welsh or Scotland or Brit* or Scot* or English or Gibraltar * or Gree* or Greenland* or Guam* or Chamorros or Hong Kong* or Croatia* or Hungar* or "Isle of Man" or Manx or Ireland or Irish or Iceland* or Israel* or Ital* or Japan* or "St Kitts" or Korea* or Kuwait* or Liechtenstein* or Lithuania* or Luxembourg* or Latvia* or Maca* or "St Martin*" or "Sint Maarten*" or Monaco or Monegasqu* or Malta or Maltese or "Northern Mariana Island*" or "New Caledonia*" or Netherlands or Dutch or Norw* or Nauru* or "New Zealand" or Kiwi or Oman* or Palau* or Poland or Polish or "Puerto Ric*" or Portug*

or "French Polynesia*" or Qatar* or "Saudi Arabia*" or Singapor* or "San Marino" or "Sammarinese" or Slovak* or Slovenia* or Sweden or Swedish or Seychell* or Creole or "Turks Caicos" or Trinidad* or Tobago* or Uruguay* or "United States" or USA or America* or Venezuela* or "Virgin Island*" or EU or "European Union" or European*).ti,ab. 3264235

2 developed countries/ or european union/ 38606

3 developed countr*.mp. 59651

4 (high income countr* or high-income countr*).mp. 11176

5 industrialised countr*.mp. 1502

6 more economically developed countr*.mp. 36

7 MEDC.mp. 43

8 advanced countr*.mp. 492

9 advanced econom*.mp. 233

10 developed econom*.mp. 455

11 industrialized econom*.mp. 43

12 industrialised econom*.mp. 11

13 (Migrant* or refugee* or immigrant* or foreigner* or newcomer* or new-comer* or migration or immigration or emigrant* or nonnative* or non-native* or diaspora or border crossing* or transient* or asylum-seek*).mp. 782544

14 ((displaced or undocumented or foreign* or resettle* or settle* or displace*) adj3 (person* or people)).mp. 4461

15 exp Human Migration/ 27663

16 Refugees/ 12718

17 "Transients and Migrants"/ 13851

18 13 or 14 or 15 or 16 or 17 784919

19 (pregnan* or postpartum or post-partum or postnatal or post-natal or antenatal or ante-natal or prenatal or pre-natal or antepartum or ante-partum or peripartum or peri-partum or birth* or trimester* or mother* or mum or mums or mom or moms or perinatal or peri-natal or childbirth* or obstetric* or puerper* or maternity or maternal).mp. 1856432

20 exp peripartum period/ or exp postpartum period/ or exp pregnancy/ 1013266

21 19 or 20 1878022

22 (intervention* or prevent* or program* or strateg* or class* or special*).mp. 7862429

23 (Peer-led or "peer led" or doula* or midwi*).mp. 41688

24 ((antenatal or Postnatal) and (session* or lesson* or workshop*)).mp. 1994

25 22 or 23 or 24 7888583

26 1 and 18 and 21 and 25 4520

27 2 or 3 or 4 or 5 or 6 or 7 or 8 or 9 or 10 or 11 or 12 89366

28 18 and 21 and 27 1198

29 26 or 28 5360

Web of Science Search Strategy

Database: Web of Science Core Collection

Entitlements:

- WOS.IC: 1993 to 2022
- WOS.CCR: 1985 to 2022
- WOS.SCI: 1900 to 2022
- WOS.AHCI: 1975 to 2022
- WOS.BHCI: 2005 to 2022
- WOS.BSCI: 2005 to 2022
- WOS.ESCI: 2015 to 2022
- WOS.ISTP: 1990 to 2022
- WOS.SSCI: 1900 to 2022
- WOS.ISSHP: 1990 to 2022

Searches:

1: TS=(Aruba* OR Andorra* OR "United Arab Emirates" OR UAE OR Emirati* OR Antigua* OR Barbuda* OR Australia* OR Austria* OR Belgi* OR Bahrain* OR Baham* OR Bermuda* OR Barbad* OR Brunei* OR Canada* OR Switzerland OR Swiss OR "Channel Island*" OR Chile OR Chilean* OR Curacao* OR "Cayman Island*" OR Caymanian* OR Cypr* OR Czech* OR German* OR Denmark OR Danish OR Spain* OR Estonia* OR Finland OR Finnish OR France OR French OR Faroe* OR "United Kingdom" OR UK OR England OR Wales OR welsh OR Scotland OR Brit* OR Scot* OR English OR "Gibraltar *" OR Gree* OR Greenland* OR Guam* OR Chamorros OR "Hong Kong*" OR Croatia* OR Hungar* OR "Isle of Man" OR Manx OR Ireland OR Irish OR Iceland* OR Israel* OR Ital* OR Japan* OR "St Kitts" OR Korea* OR Kuwait* OR Liechtenstein* OR Lithuania* OR Luxembourg* OR Latvia* OR Maca* OR "St Martin*" OR "Sint Maarten*" OR Monaco OR Monegasqu* OR Malta OR Maltese OR "Northern Mariana Island*" OR "New Caledonia*" OR Netherlands OR Dutch OR Norw* OR Nauru* OR "New Zealand" OR Kiwi OR Oman* OR Palau* OR Poland OR Polish OR "Puerto Ric*" OR Portug* OR "French Polynesia*" OR Qatar* OR "Saudi Arabia*"

OR Singapor* OR "San Marino" OR Sammarinese OR Slovak* OR Slovenia* OR Sweden OR Swedish OR Seychell* OR Creole OR "Turks Caicos" OR Trinidad* OR Tobago* OR Uruguay* OR "United States" OR USA OR America* OR Venezuela* OR "Virgin Island*" OR EU OR "European Union" OR European* OR "high income countr*" OR "high-income countr*" OR "industrialised countr*" OR "more economically developed countr*" OR "advanced countr*" OR "advanced econom*" OR "MEDC" OR "developed econom*" OR "industrialized econom*" OR "industrialised econom*")

Date Run: Mon Dec 12 2022 13:10:27 GMT+0000 (Greenwich Mean Time)
Results: 10269737

2: TS=(migrant* OR refugee* OR immigrant* OR foreigner* OR newcomer* OR new-comer* OR migration OR immigration OR emigrant* OR nonnative* OR non-native* OR diaspora OR "border crossing*" OR transient* OR asylum-seek* OR "Displaced people*" OR "undocumented people*" OR "foreign* people*" OR "resettle* people*" OR "settle* people*" OR "Displaced person*" OR "undocumented person*" OR "foreign* person*" OR "resettle* person*" OR "settle* person*")

Date Run: Mon Dec 12 2022 13:12:33 GMT+0000 (Greenwich Mean Time)
Results: 1534587

3: TS=(pregnan* OR postpartum OR post-partum OR postnatal OR post-natal OR antenatal OR ante-natal OR prenatal OR pre-natal OR antepartum OR ante-partum OR peripartum OR peri-partum OR birth* OR trimester* OR mother* OR mum OR mums OR mom OR moms OR perinatal OR peri-natal OR childbirth* OR obstetric* OR puerper* OR maternity OR maternal)

Date Run: Mon Dec 12 2022 13:13:05 GMT+0000 (Greenwich Mean Time)
Results: 1676056

4: TS=((intervention* OR prevent* OR program* OR strateg* OR class* OR special* OR peer-led OR "peer led" OR doula* OR midwi*) OR ((antenatal OR postnatal) AND (class* OR session* OR lesson* OR workshop*)))

Date Run: Mon Dec 12 2022 13:13:46 GMT+0000 (Greenwich Mean Time)
Results: 12259143

5: #4 AND #3 AND #2 AND #1

Date Run: Mon Dec 12 2022 13:13:54 GMT+0000 (Greenwich Mean Time)
Results: 5662

APA PsycInfo <1806 to December Week 1 2022>

1 (Aruba* or Andorra* or "United Arab Emirates" or UAE or Emirati* or Antigua* or Barbuda* or Australia* or Austria* or Belgi* or Bahrain* or Baham* or Bermuda* or Barbad* or Brunei* or Canada* or Switzerland or Swiss or "Channel Island*" or Chile or Chilean* or Curacao* or "Cayman Island*" or Caymanian* or Cypr* or Czech* or German* or Denmark or Danish or Spain* or Estonia* or Finland or Finnish or France or French or Faroe* or "United Kingdom" or UK or England or Wales or welsh or Scotland or Brit* or Scot* or English or Gibraltar * or Gree* or Greenland* or Guam* or Chamorros or Hong Kong* or Croatia* or Hungar* or "Isle of Man" or Manx or Ireland or Irish or Iceland* or Israel* or Ital* or Japan* or "St Kitts" or Korea* or Kuwait* or Liechtenstein* or Lithuania* or Luxembourg* or Latvia* or Maca* or "St Martin*" or "Sint Maarten*" or Monaco or Monegasqu* or Malta or Maltese or "Northern Mariana Island*" or "New Caledonia*" or Netherlands or Dutch or Norw* or Nauru* or "New Zealand" or Kiwi or Oman* or Palau* or Poland or Polish or "Puerto Ric*" or Portug* or "French Polynesia*" or Qatar* or "Saudi Arabia*" or Singapor* or "San Marino" or "Sammarinese" or Slovak* or Slovenia* or Sweden or Swedish or Seychell* or Creole or "Turks Caicos" or Trinidad* or Tobago* or Uruguay* or "United States" or USA or America* or Venezuela* or "Virgin Island*" or EU or "European Union" or European*).ti,ab. 998036

2 developed countries/ 1444

3 developed countr*.mp. 6366

4 (high income countr* or high-income countr*).mp. 2276

5 industrialised countr*.mp. 162

6 more economically developed countr*.mp. 9

7 MEDC.mp. 4

8 advanced countr*.mp. 149

9 advanced econom*.mp. 164

10 developed econom*.mp. 377

11 industrialized econom*.mp. 54

12 industrialised econom*.mp. 3

13 (Migrant* or refugee* or immigrant* or foreigner* or newcomer* or new-comer* or migration or immigration or emigrant* or nonnative* or non-native* or diaspora or border crossing* or transient* or asylum-seek*).mp. 106941

14 ((displaced or undocumented or foreign* or resettle* or settle* or displace*) adj3 (person* or people)).mp. 1523

15 exp Human Migration/ 17211

16 13 or 14 or 15 108706

17 (pregnan* or postpartum or post-partum or postnatal or post-natal or antenatal or ante-natal or prenatal or pre-natal or antepartum or ante-partum or peripartum or peri-partum or birth* or trimester* or mother* or mum or mums or mom or moms or perinatal or peri-natal or childbirth* or obstetric* or puerper* or maternity or maternal).mp. 286207

18 exp Intrapartum Period/ or exp Antepartum Period/ or Perinatal Period/ or exp pregnancy/ or Postnatal Period/ or exp Birth/ or exp Prenatal Care/ 54584

19 17 or 18 287131

20 (intervention* or prevent* or program* or strateg* or class* or special*).mp. 1754113

21 (Peer-led or "peer led" or doula* or midwi*).mp. 5292

22 ((antenatal or Postnatal) and (session* or lesson* or workshop*)).mp. 857

23 20 or 21 or 22 1756811

24 1 and 16 and 19 and 23 1693

25 2 or 3 or 4 or 5 or 6 or 7 or 8 or 9 or 10 or 11 or 12 9141

26 16 and 19 and 25 69

27 24 or 26 1739

Cochrane Central Register of Controlled Trials

Issue 11 of 12, November 2022

Search Name: CL

Date Run: 22/12/2022 10:48:39

Comment:

ID	Search	Hits
#1	MeSH descriptor: [Developed Countries] explode all trees	52
#2	MeSH descriptor: [European Union] explode all trees	65
#3	(developed countr*):ti,ab,kw	4180
#4	(high income countr*):ti,ab,kw	2009
#5	(high-income countr*):ti,ab,kw	854
#6	(industrialised countr*):ti,ab,kw	459
#7	(economically developed countr*):ti,ab,kw	35
#8	(MEDC):ti,ab,kw	2
#9	(advanced countr*):ti,ab,kw	1500
#10	(advanced econom*):ti,ab,kw	957
#11	(developed econom*):ti,ab,kw	3125
#12	(industrialized econom*):ti,ab,kw	62

#13 (industrialised econom*):ti,ab,kw 62

#14 {OR #1-#13} 10948

#15 (Migrant*):ti,ab,kw 441

#16 (refugee):ti,ab,kw 444

#17 (immigrant*):ti,ab,kw 839

#18 (foreigner):ti,ab,kw 21

#19 (newcomer*):ti,ab,kw 48

#20 (new-comer*):ti,ab,kw 5

#21 (migration):ti,ab,kw 3759

#22 (immigration):ti,ab,kw 231

#23 (emigrant*):ti,ab,kw 238

#24 (nonnative*):ti,ab,kw 86

#25 (non-native*):ti,ab,kw 77

#26 (diaspora):ti,ab,kw 4

#27 (border crossing*):ti,ab,kw 16

#28 (transient*):ti,ab,kw 19243

#29 (asylum-seek*):ti,ab,kw 82

#30 ((displaced or undocumented or foreign* or resettle* or settle* or displace*) NEAR/3 (person* or people)):ti,ab,kw 89

#31 MeSH descriptor: [Human Migration] explode all trees 68

#32 MeSH descriptor: [Refugees] this term only 173

#33 MeSH descriptor: [Transients and Migrants] this term only 80

#34 {OR #15-#33} 24729

#35 (pregnan* or postpartum or post-partum or postnatal or post-natal or antenatal or ante-natal or prenatal or pre-natal or antepartum or ante-partum or peripartum or peri-partum or birth* or trimester* or mother* or mum or mums or mom or moms or perinatal or peri-natal or childbirth* or obstetric* or puerper* or maternity or maternal):ti,ab,kw 119742

#36 MeSH descriptor: [Peripartum Period] explode all trees 21

#37 MeSH descriptor: [Postpartum Period] explode all trees 1942

#38 MeSH descriptor: [Pregnancy] explode all trees 25029

#39 {OR #35-#38} 119890

#40 #14 AND #34 AND #39 54

Interface - EBSCOhost Research Databases
Search Screen - Advanced Search
Database - CINAHL Plus

#	Query	Limiters/Expanders	Results
S1	MH "developed countries"	Search modes - Boolean/Phrase	4,144
S2	(MH "European Union")	Search modes - Boolean/Phrase	6,324
S3	TX developed countr*	Search modes - Boolean/Phrase	14,015
S4	TX high income countr*	Search modes - Boolean/Phrase	5,938
S5	TX high-income countr*	Search modes - Boolean/Phrase	5,253
S6	TX industrialised countr*	Search modes - Boolean/Phrase	409
S7	TX economically developed countr*	Search modes - Boolean/Phrase	113
S8	TX MEDC	Search modes - Boolean/Phrase	9
S9	TX advanced countr*	Search modes - Boolean/Phrase	564
S10	TX advanced econom*	Search modes - Boolean/Phrase	406
S11	TX developed econom*	Search modes - Boolean/Phrase	1,039
S12	TX industrialized econom*	Search modes - Boolean/Phrase	50
S13	TX industrialised econom*	Search modes - Boolean/Phrase	11
S14	S1 OR S2 OR S3 OR S4 OR S5 OR S6 OR S7 OR S8 OR S9 OR S10 OR S11 OR S12 OR S13	Search modes - Boolean/Phrase	27,284
S15	TX Migrant* or refugee* or immigrant* or foreigner* or	Search modes - Boolean/Phrase	106,229

	newcomer* or new-comer* or migration or immigration or emigrant* or nonnative* or non-native* or diaspora or border crossing* or transient* or asylum-seek*		
S16	TX (displaced or undocumented or foreign* or resettle* or settle* or displace*) n3 (person* or people)	Search modes - Boolean/Phrase	1,998
S17	(MH "Refugees+") OR (MH "Transients and Migrants")	Search modes - Boolean/Phrase	14,390
S18	S15 OR S16 OR S17	Search modes - Boolean/Phrase	107,366
S19	TX pregnan* or postpartum or post-partum or postnatal or post-natal or antenatal or ante-natal or prenatal or pre-natal or antepartum or ante-partum or peripartum or peri-partum or birth* or trimester* or mother* or mum or mums or mom or moms or perinatal or peri-natal or childbirth* or obstetric* or puerper* or maternity or maternal	Search modes - Boolean/Phrase	502,585
S20	(MH "Pregnancy+")	Expanders - Apply equivalent subjects Search modes - Boolean/Phrase	246,130
S21	(MH "Postnatal Period+")	Expanders - Apply equivalent subjects Search modes - Boolean/Phrase	16,885

S22	(MH "Postnatal care+") OR (MH "Prenatal Care") OR (MH "Perinatal Care")	Search modes - Boolean/Phrase	29,500
S23	S19 OR S20 OR S21 OR S22	Search modes - Boolean/Phrase	504,522
S24	S14 AND S18 AND S23	Search modes - Boolean/Phrase	237
S25	TI (Aruba* or Andorra* or "United Arab Emirates" or UAE or Emirati* or Antigua* or Barbuda* or Australia* or Austria* or Belgi* or Bahrain* or Baham* or Bermuda* or Barbad* or Brunei* or Canada* or Switzerland or Swiss or "Channel Island*" or Chile or Chilean* or Curacao* or "Cayman Island*" or Caymanian* or Cypr* or Czech* or German* or Denmark or Danish or Spain* or Estonia* or Finland or Finnish or France or French or Faroe* or "United Kingdom" or UK or England or Wales or welsh or Scotland or Brit* or Scot* or English or Gibraltar * or Gree* or Greenland* or Guam* or Chamorros or Hong Kong* or Croatia* or Hungar* or "Isle of Man" or Manx or Ireland or Irish or Iceland* or Israel* or Ital* or Japan* or "St Kitts" or Korea* or Kuwait* or Liechtenstein* or	Search modes - Boolean/Phrase	1,194,855

	Lithuania* or Luxembourg* or Latvia* or Maca* or "St Martin*" or "Sint Maarten*" or Monaco or Monegasqu* or Malta or Maltese or "Northern Mariana Island*" or "New Caledonia*" or Netherlands or Dutch or Norw* or Nauru* or "New Zealand" or Kiwi or Oman* or Palau* or Poland or Polish or "Puerto Ric*" or Portug* or "French Polynesia*" or Qatar* or "Saudi Arabia*" or Singapor* or "San Marino" or "Sammarinese" or Slovak* or Slovenia* or Sweden or Swedish or Seychell* or Creole or "Turks Caicos" or Trinidad* or Tobago* or Uruguay* or "United States" or USA or America* or Venezuela* or "Virgin Island*" or EU or "European Union" or European*) OR AB (Aruba* or Andorra* or "United Arab Emirates" or UAE or Emirati* or Antigua* or Barbuda* or Australia* or Austria* or Belgi* or Bahrain* or Baham* or Bermuda* or Barbad* or Brunei* or Canada* or Switzerland or Swiss or "Channel Island*" or Chile or Chilean* or Curacao* or		

	"Cayman Island*" or Caymanian* or Cypr* or Czech* or German* or Denmark or Danish or Spain* or Estonia* or Finland or Finnish or France or French or Faroe* or "United Kingdom" or UK or England or Wales or welsh or Scotland or Brit* or Scot* or English or Gibraltar * or Gree* or Greenland* or Guam* or Chamorros or Hong Kong* or Croatia* or Hungar* or "Isle of Man" or Manx or Ireland or Irish or Iceland* or Israel* or Ital* or Japan* or "St Kitts" or Korea* or Kuwait* or Liechtenstein* or Lithuania* or Luxembourg* or Latvia* or Maca* or "St Martin*" or "Sint Maarten*" or Monaco or Monegasqu* or Malta or Maltese or "Northern Mariana Island*" or "New Caledonia*" or Netherlands or Dutch or Norw* or Nauru* or "New Zealand" or Kiwi or Oman* or Palau* or Poland or Polish or "Puerto Ric*" or Portug* or "French Polynesia*" or Qatar* or "Saudi Arabia*" or Singapor* or "San Marino" or "Sammarinese" or Slovak* or Slovenia* or Sweden or		

	Swedish or Seychell* or Creole or "Turks Caicos" or Trinidad* or Tobago* or Uruguay* or "United States" or USA or America* or Venezuela* or "Virgin Island*" or EU or "European Union" or European*)		
S26	S18 AND S23 AND S25	Search modes - Boolean/Phrase	4,466
S27	TX intervention* or prevent* or program* or strateg* or "classes" or "specialist" OR "specialists" OR Peer-led or "peer led" or doula* or midwi*	Search modes - Boolean/Phrase	2,112,926
S28	TX (antenatal or Postnatal) AND (session* or lesson* or workshop*)	Search modes - Boolean/Phrase	1,184
S29	S27 OR S28	Search modes - Boolean/Phrase	2,113,168
S30	S26 AND S29	Search modes - Boolean/Phrase	1,960
S31	S24 OR S30	Search modes - Boolean/Phrase	2,128

Grey Literature and Registry Searching:

WHO International Clinical Trials Registry Platform (ICTRP):

https://trialsearch.who.int/AdvSearch.aspx using Pregnancy AND migrant

Clinicaltrials.gov:

https://clinicaltrials.gov/ct2/search/advanced?cond=&term=&cntry=&state=&city=&dist=
using the terms ‘Pregnancy’ ‘Migrant’ in the 'other terms' search field

Google Scholar:

Pregnancy refugee OR migrant OR asylum "high income"

World Health Organization Website:

site:who.int pregnancy refugee OR migrant OR asylum "high income":
https://www.google.com/search?q=site%3Awho.int+pregnancy+refugee+OR+migrant+OR+asylum+%22high+income%22

UN Refugee Agency Website:

site:unhcr.org pregnancy refugee OR migrant OR asylum "high income":
https://www.google.com/search?q=site%3Aunhcr.org+pregnancy+refugee+OR+migrant+OR+asylum+%22high+income%22
