# Supplementary material for: Interventions to improve perinatal outcomes among migrant women in high-income countries: a systematic review protocol
Source: BMJ Open. 2023 Aug 17;13(8):e072090. doi: 10.1136/bmjopen-2023-072090 (PMC10441090; doi:10.1136/bmjopen-2023-072090)
Supplement: Author's manuscript [file bmjopen-2023-072090.draft_revisions.pdf]

BMJ Open

BMJ Open is committed to open peer review. As part of this commitment we make the peer review history of every article we publish publicly available.

When an article is published we post the peer reviewers' comments and the authors' responses online. We also post the versions of the paper that were used during peer review. These are the versions that the peer review comments apply to.

The versions of the paper that follow are the versions that were submitted during the peer review process. They are not the versions of record or the final published versions. They should not be cited or distributed as the published version of this manuscript.

BMJ Open is an open access journal and the full, final, typeset and author-corrected version of record of the manuscript is available on our site with no access controls, subscription charges or pay-per-view fees (http://bmjopen.bmj.com).

If you have any questions on BMJ Open's open peer review process please email info.bmjopen@bmj.com

BMJ Open

Interventions to Improve Perinatal Outcomes amongst Migrant Women in High-Income Countries: A Systematic Review Protocol

Journal:	*BMJ Open*
Manuscript ID	bmjopen-2023-072090
Article Type:	Protocol
Date Submitted by the Author:	20-Jan-2023

Complete List of Authors:	Stevenson, Kerrie; University College London, Institute of Health Informatics<br>Ogunlana, K; University College London, Institute of Health Informatics<br>Edwards, Samuel; University of Warwick, Warwick Medical School<br>Henderson, William; University College London, Library Services<br>Rayment-Jones, Hannah; King's College London, Women and Children's Health<br>McGranahan, Majel; University of Warwick<br>Marti-Castaner, Maria; University of Copenhagen, Health Department of Public Health<br>Fellmeth, Gracia; University of Oxford Nuffield Department of Population Health<br>Luchenski, Serena; University College London, Institute of Health Informatics<br>Stevenson, Fiona; University College London, Primary Care and Population Sciences<br>Knight, Marian; University of Oxford, National Perinatal Epidemiology Unit<br>Aldridge, Robert; University College London, Institute of Health Informatics

Keywords:	OBSTETRICS, PUBLIC HEALTH, PAEDIATRICS

SCHOLARONE™
Manuscripts

Interventions to Improve Perinatal Outcomes amongst Migrant Women in High-Income Countries: A Systematic Review Protocol

Authors

K Stevenson[1*], K Ogunlana[1], S Edwards[2], W Henderson[3], H Raymont-Jones[4], M McGranahan[2], M Marti-Castaner[5], G Fellmeth[6], S Luchenski[7], F Stevenson[8], M Knight[5], R Aldridge[1]

Affiliations

1. Institute of Health Informatics, University College London, 222 Euston Road, London, NW1 2DA, UK.
2. Warwick Medical School, University of Warwick, Coventry, CV4 7HL, UK.
3. Library Services, University College London, Gower Street, London, WC1E 6BT, UK.
4. Department of Women and Children's Health, King's College London, Strand, London, WC2R 2LS, UK.
5. Danish Research Centre for Migration, Ethnicity, and Health, Department of Public Health, University of Copenhagen, Øster Farimagsgade 5,1014 København K, Denmark.
6. National Perinatal Epidemiology Unit, Nuffield Department of Population Health, University of Oxford, Old Road Campus, Headington, Oxford OX3 7LF, UK.
7. Collaborative Centre for Inclusion Health, University College London, 1-19 Torrington Place, London, WC1E 7HB UK.
8. Research Department of Primary Care and Health, Royal Free Campus, Rowland Hill Street, London, NW33 2PF, UK.

***Corresponding Author**

Dr Kerrie Stevenson

Email: k.stevenson@ucl.ac.uk

Address: Institute of Health Informatics, University College London, 222 Euston Road, London, NW1 2DA

ABSTRACT

Introduction

Women who are migrants and who are pregnant or postpartum are at high risk of poorer perinatal outcomes compared to host country populations due to experiencing numerous additional stressors including social exclusion and language barriers. High-income countries (HICs) host many migrants, including forced migrants who may face additional challenges in the peripartum period. Although HICs' maternity care systems are often well developed, they are not routinely tailored to the needs of migrant women. The primary outcome of this systematic review is to provide an assessment of what interventions exist to improve perinatal outcomes in HICs. The secondary outcome is to assess the most effective interventions for improving perinatal outcomes in HICs.

Methods and Analysis

This protocol follows the Preferred Reporting Items for Systematic Reviews and Meta-Analyses Protocols guidelines. EMBASE, EMCARE, MEDLINE and PsycInfo, CENTRAL, Scopus, CINAHL Plus, and Web of Science, as well as grey literature sources will be searched from inception up until December 2022. We will include randomised-controlled trials, quasi-experimental and interventional studies of interventions which aim to improve perinatal outcomes in any HIC. There will be no language restrictions. We will exclude studies presenting only qualitative outcomes and those including mixed populations of migrant and non-migrant women. Screening and data extraction will be completed by two independent reviewers and risk of bias will be assessed using the Quality Assessment Tool for Quantitative Studies. If a collection of suitably comparable outcomes is retrieved, we will perform meta-analysis applying a random effects model. Presentation of results will comply with guidelines in the Cochrane Handbook of Systematic Reviews of Interventions and the PRISMA statement.

Ethics and Dissemination

For peer review only

Ethical approval is not required. Results will be submitted for peer-reviewed publication and presented at national and international conferences. The findings will inform the work of the Lancet Migration European Hub.

PROSPERO Registration Number

CRD42022380678

Strengths and Limitations of this Study

- We aim to complete what we believe is the first systematic review of interventions to improve perinatal outcomes for migrant women in high-income settings.
- We will employ rigorous methodology in accordance with the Cochrane Handbook of Systematic Reviews. The results will be reported in accordance with the Preferred Reporting Items for Systematic Reviews and Meta-Analyses (PRISMA) statement, and we aim to assess if included studies employed co-production methods at any point in intervention development or analysis.
- The search strategy was developed with an experienced medical librarian and adapted for each database searched.
- No language restrictions will be employed, and we are doing an extensive and systematic grey literature search which is often omitted in similar reviews.
- The certainty of evidence may be limited by the number of studies available and because some studies may be of low quality without a quantitative outcome assessment.

For peer review only

BACKGROUND

Rationale

There are an estimated 281 million international migrants in the world, constituting 3.6% of the global population.[1] Of these, approximately 13% are forced migrants, including refugees and asylum seekers who often face significant hardships during migration and when settled in their host country.[2] The remainder are economic migrants who often choose to migrate to reunite with family or for better job prospects, but they may also experience marginalisation due to poverty, social isolation, and discrimination.[1] Between 2000 and 2022 the international migrant population increased by 108 million, and although the majority of international migrants originate from and within low- and middle-income countries (LMICs), increasing numbers hope to settle in high-income countries (HICs).[1] HICs are not always well adapted to care for the needs of marginalised migrants, and many HICs struggle to adapt to this changing migration landscape.[1]

Women constitute approximately half of the international migrant population.[2] Women who migrate and who are pregnant or post-partum can face significant barriers to accessing maternity care, putting them at increased risk of poor mental and physical health.[3-6] They may experience poor social support in their host country, an inability to access to healthcare, and language barriers.[5,7] Forced migrant women who are pregnant or post-partum experience additional challenges including pre-migration stressors such as the trauma of war, transition stressors including dangerous migration journeys and gender-based violence, and post-migration stressors such as poor access to legal entitlements, discrimination, and socio-cultural barriers in obstetric care.[7,8] They are also more likely to have poor access to antenatal care and experience higher rates of perinatal mortality, miscarriage, and stillbirth than non-refugee women.[9] HICs often have some of the resources needed to support marginalised pregnant and postpartum migrant women, but often struggle to provide optimum care.[8]

For peer review only - http://bmjopen.bmj.com/site/about/guidelines.xhtml

To our knowledge no previous review has sought to quantitatively synthesise the literature on the most effective interventions to improve perinatal outcomes for migrant women in HICs. Balaam et al. conducted a systematic review in 2020 which aimed to identify social support interventions for asylum-seeking and refugee women in Europe.[10] The findings were qualitatively synthesised and women valued peer support and interventions that addressed their needs in a holistic way.[10] The UK National Institute of Health and Care Research (NIHR) commissioned a systematic review to explore interventions to improve maternity care for migrant women in the UK in 2017.[11] The review included only UK studies and identified just four interventions. These included peer support and specialist maternity care interventions, but they were not robustly evaluated so it was difficult to draw conclusions on their effectiveness.[11]

Objectives

This systematic review aims to explore the most effective interventions to improve perinatal outcomes for migrant women in HICs by quantitatively synthesising the literature. The primary outcome will be to determine what interventions exist to improve perinatal outcomes for migrant women in HICs. The secondary outcome will be to explore the effectiveness of these interventions.

METHODS/DESIGN

Registration and Protocol Adherence

This systematic review will be reported in accordance with the Preferred Reporting Items for Systematic Reviews and Meta-Analyses (PRISMA) guidelines and was registered on PROSPERO: CRD42022380678 on 9th December 2022.

Definitions

For the purposes of this review, migrant women will be defined as being aged 16 years or older and who were born outside their host country. Eligible maternity care interventions are

For peer review only

any hospital- or community-based initiatives offered in the antenatal, perinatal, or postnatal period up to one year postpartum. The perinatal period is defined as pregnancy and up to one year postpartum.

Eligibility Criteria and Patient, Intervention, Comparison Outcome (PICO) Framework

PICO Framework

- Population: Perinatal migrant women (those who were not born in their host country) aged 16 years or older and living in HICs (defined as being in the World Bank high-income economy category).[12]
- Intervention: Any hospital- or community-based activity undertaken with the aim of improving perinatal outcomes and delivered during the antenatal period and up to one year postpartum.
- Control: Usual care, if data are available.
- Outcome: Improvements in perinatal outcomes (rates of miscarriage, preterm birth, stillbirth, birthweight, mode of delivery, APGAR score, maternal/neonatal critical care admission, breastfeeding initiation and duration, maternal/neonatal death, perinatal mental illness); number of antenatal or postnatal appointments attended; or change in maternal wellbeing as assessed by validated mental illness or wellbeing screening scales. The main outcomes of interest will be rates of pre-term birth, birthweight, and number of antenatal or postnatal appointments attended as these are key mediators of longer-term health of the infant and crucial measures of quality of maternity care in accordance with the World Health Organisation guidelines for Quality of Care for Pregnant Women and Newborns.[13]

Inclusion and Exclusion Criteria

Observational, quasi-experimental, and experimental intervention studies published from inception will be included. Abstracts, non-empirical research, opinion, or editorial pieces will be excluded. If duplicate reports or publications of the same data are retrieved, the less

For peer review only

complete or recent version will be excluded if the same data are reported. Studies including only a qualitative outcome assessment will be excluded. Interventions that were not specifically designed or adapted for migrant women in the perinatal period will be excluded.

Search Strategy and Data Sources

EMBASE, EMCARE, MEDLINE and PsycInfo via Ovid, Cochrane Central Register of Controlled Trials (CENTRAL) via Cochrane Library, Scopus, CINAHL Plus via EBSCOHost, and Web of Science from inception up until December 2022. Grey literature sources including Google Scholar and trial registries were searched in December 2022. The first 150 results from the following supplementary sources will be searched: Google Scholar, WHO International Clinical Trials Registry Platform (ICTRP), Clinicaltrials.gov, the World Health Organisation Website, and the UN Refugee Agency website. Forward citation searching will be employed on all included articles, and the reference lists of all included articles will also be searched. No language restrictions will be employed. If studies are published in a language other than English, one of the research team who is fluent in that language will assess for likely relevance and extract the data, if appropriate. The article will also be translated using Google Translate™ and a second reviewer will review its relevance and extract the data, if appropriate. If none of the research team are fluent in the language, we will pay UCL's graduate linguistics programme to translate article. The Boolean operators "AND" and "OR" will be employed to combine the descriptors. An experienced medical librarian helped to develop the search strategy and it has been adapted for each database. A preliminary search strategy for Medline is provided in Appendix 1. EndNote™ will be used to collect and manage studies retrieved.[14] Covidence™ will be used for deduplication and for study selection.[15]

Data Extraction (Selection and Coding)

Two independent reviewers will screen the titles and abstracts of all the citations for relevance. Full text manuscripts for relevant articles will be obtained. Full texts will be

For peer review only

independently assessed for eligibility using a checklist of the inclusion and exclusion criteria by two independent reviewers. Studies meeting the inclusion criteria will be selected for inclusion in the review. All excluded articles from the full text screening will be retained with reason for exclusion noted. Disagreements between reviewers will be discussed and agreement sought from a third reviewer if necessary. If data are not accessible from the paper, the authors will be contacted. All extracted data will be recorded on the piloted data extraction form by two separate reviewers and cross-checked. The main data fields will be: (a) Author, publication year; (b) Country; (c) Study design; (d) Population and baseline characteristics; (e) Context (community or hospital-based or online); (f) Intervention details; (g) Control or comparison; (h) Timing of outcome measurements; (i) Outcome measures (type, scale used, scale validation status); (j) Outcomes; (k) Quality assessment; (l) Reported according to CONSORT guidelines; (m) Co-production methods used. A flowchart will summarise the selection process in line with the PRISMA 2020 guidelines. Study characteristics will be summarised and presented in tables.

Quality Assessment / Risk of Bias

Two reviewers will perform the critical appraisal independently and this will be independently cross-checked. The Quality Assessment Tool for Quantitative Studies will be used to assess rigor for each included study.[16] This was chosen as it has been developed and validated to assess both observational and experimental studies and shows reliability and validity. It assesses selection bias, study design, confounders, blinding, data collection, withdrawals, intervention integrity, and statistical analysis. If meta-analysis is conducted, publication bias will be assessed using a funnel plot.

Data Synthesis

Study characteristics including outcomes e.g., raw proportions, mean scores, and odds/risk ratios will be extracted and presented in tabular form. Narrative synthesis will be conducted according to Cochrane guidance and will include the creation of categories of interventions

For peer review only

based on included papers e.g. specialist midwifery services, interpreting services, and use of a doula.[17,18] If appropriate, heterogeneity between studies will be explored using the I^2 statistic. Random effects meta-analysis will be conducted with 95% confidence intervals to allow for expected heterogeneity between different study populations, if appropriate. If possible, pooled estimates of odds/risk ratios with 95% confidence intervals will be calculated to explore outcomes among migrant women compared to usual care. Subgroup analyses according to migration status (economic and forced) and study context (hospital- or community-based) will be conducted, if appropriate. Sensitivity analysis according to study quality and method of recruitment will be undertaken. If meta-analysis is not possible due to a lack of standardised outcome data, alternative quantitative methods will be used following the Cochrane Handbook for Systematic Reviews of Interventions guidance.[18] The handbook details several approaches, but it is likely a Harvest Plot will be most appropriate. A Harvest Plot provides a visual extension of vote counting by categorising studies based on their effect (e.g. 'beneficial effect' or 'detrimental effect').[19] Vote counting is recommended when there are inconsistent effect measures across studies. Traditional vote counting methods using statistical significance, magnitude of effect, or subjective rules have been shown to be misleading.[18] Instead, we will create a standardised binary metric, 'beneficial' or 'detrimental' based on direction of effect for each intervention according to outcome category (e.g. birthweight, stillbirth rates, or antenatal appointment attendance).[18] We will calculate the proportion of beneficial studies, 95% confidence interval (binomial exact calculation), and p-value (binomial probability test) to demonstrate if there is any evidence of an effect. We will present the findings on a Harvest Plot which will take the form of a 'supermatrix' which visually displays the vote counting results for each intervention.[18] All statistical analyses will be conducted using R Studio.[20]

Confidence in Cumulative Evidence

For peer review only

Confidence in the strength of evidence found will be assessed using the overall assessment outlined in the Quality Assessment Tool for Quantitative Studies checklist.[16] This will be done by two reviewers and possible disagreement will be assessed by third reviewer.

DISCUSSION

This systematic review will provide a comprehensive overview of interventions being used to improve perinatal outcomes for migrant women in HICs. It will make a systematic assessment of the most effective interventions which will help to inform policy and clinical decision-making across these regions. The findings can also be used by researchers planning or adapting maternity care interventions for migrant women in HICs, as well as to guide resource allocation decisions by funders and healthcare managers. Potential limitations include the retrieval of low- quality studies with poor evaluation of outcomes which will inhibit our ability to robustly assess the evidence of effectiveness of these interventions. Attempts to retrieve all available literature have been utilised such as involving a medical librarian in the development of our search strategy to ensure a broad and sensitive selection of search terms and searching multiple databases and additional supplementary and grey literature sources, however it is possible that some relevant studies/data may be missed. We are taking a co-production approach to the planning, conducting, and interpretation of this work by ensuring migrant women who have given birth or been pregnant in their host country are involved throughout. We will also be assessing if included studies have taken a co-production approach at any point in intervention development, implementation, or evaluation to gain an understanding of the prevalence of co-production in this field. We will identify gaps in the evidence to guide future research priorities.

Ethics and Dissemination

Ethical approval is not required for this study as no primary data are being collected. We intend to publish the results in a peer-reviewed open access publication and present results

For peer review only

at national and international conferences. The findings will inform the work of the Lancet Migration European Regional Hub.

Patient and Public Involvement

Migrant women who are residing in the UK helped with the review's inception and design, and will also help with data extraction, analysis, and interpretation. They will also help with disseminating the work through co-authorship on peer-reviewed manuscripts and presentations at conferences.

Authors' Contributions

KS, KO, FS, MN, RA conceived of the review. KS, KO, SE, WH, HRJ, MM, MMC, GF, SL, FS, MN, RA developed the approach and protocol. KS developed the search strategy. KS wrote the first draft of the protocol, and all authors were invited to comment. KS is the guarantor of the review. All authors approved the final version of the protocol.

Funding Statement

KS is supported by a National Institute of Health and Care Research (NIHR) Doctoral Fellowship [Grant number NIHR302577]. GF is funded by a Clinical Research Fellowship from the Nuffield Department of Population Health, University of Oxford. MM is supported by a Medical Research Council Clinical Research Training Fellowship [Grant number MR/W01498X/1]. The funders played no part in the design and writing of the protocol.

Competing Interests Statement

None declared.

Word Count

2018.

For peer review only

References

1. UN Migration. *World Migration Report 2022*. 2022. https://www.iom.int/wmr/interactive

2. UN Refugee Agency. Figures at a glance. https://www.unhcr.org/uk/figures-at-a-glance.html

3. Sudbury H, Robinson A. Barriers to sexual and reproductive health care for refugee and asylum-seeking women. Article. *British Journal of Midwifery*. 2016;24(4):275-281. doi:10.12968/bjom.2016.24.4.275

4. Fair F, Raben L, Watson H, Vivilaki V, Muijsenbergh Mvd, Soltani H. Migrant women's experiences of pregnancy, childbirth and maternity care in European countries: a systematic review. *PLoS ONE*. 2020;15(2)doi:http://dx.doi.org/10.1371/journal.pone.0228378

5. Almeida LM, Caldas J, Ayres-De-Campos D, Salcedo-Barrientos D, Dias S. Maternal healthcare in migrants: A systematic review. Review. *Maternal and Child Health Journal*. 2013;17(8):1346-1354. doi:10.1007/s10995-012-1149-x

6. Anderson FM, Hatch SL, Comacchio C, Howard LM. Prevalence and risk of mental disorders in the perinatal period among migrant women: a systematic review and meta-analysis. *Arch Womens Ment Health*. 2017;20(3):449-62. doi:10.1007/s00737-017-0723-z

7. Fair F, Watson H, Soltani H, Raben L, Vivilaki V, van den Muijsenbergh M. Migrant women's experiences of pregnancy, childbirth and maternity care in European countries: A systematic review. *PLoS ONE*. 2020;15(2):e0228378. doi:http://dx.doi.org/10.1371/journal.pone.0228378

8. World Health Organization Regional Office for Europe. *Improving the health care of pregnant refugee and migrant women and newborn children*. 2018. https://www.euro.who.int/__data/assets/pdf_file/0003/388362/tc-mother-eng.pdf

9. Sturrock S, Williams E, Greenough A. Antenatal and perinatal outcomes of refugees in high income countries. *Journal of perinatal medicine*. 2020;49(1):80-93. doi:https://dx.doi.org/10.1515/jpm-2020-0389

10. Balaam M-C, Kingdon C, Haith-Cooper M. A Systematic Review of Perinatal Social Support Interventions for Asylum-seeking and Refugee Women Residing in Europe. ReviewPaper. *Journal of Immigrant and Minority Health*. 2021-07-17 2021;24(3):741-758. doi:doi:10.1007/s10903-021-01242-3

11. Higginbottom GMA, Evans C, Hussain B, Morgan M, Bharj KK, Eldridge J. Experience of and access to maternity care in the UK by immigrant women: A narrative synthesis systematic review. *BMJ Open*. 2019;9(12):e029478. doi:http://dx.doi.org/10.1136/bmjopen-2019-029478

12. The World Bank. World Bank Country and Lending Groups. https://datahelpdesk.worldbank.org/knowledgebase/articles/906519

13. Tunçalp Ö, Were W, MacLennan C, et al. Quality of care for pregnant women and newborns-the WHO vision. *BJOG : an international journal of obstetrics and gynaecology*. 2015 Jul 2015;122(8)doi:10.1111/1471-0528.13451

14. *EndNote*. Version 20. Clarivate; 2013.

15. *Covidence Systematic Review Software*. Veritas Health Innovation; 2021.

16. Effective Public Healthcare Panacea Project. Quality Assessment Tool for Quantitative Studies. https://www.ephpp.ca/quality-assessment-tool-for-quantitative-studies/

17. Popay J, Roberts H, Sowden A, et al. *Guidance on the Conduct of Narrative Synthesis in Systematic Reviews*. 2006.

For peer review only - http://bmjopen.bmj.com/site/about/guidelines.xhtml

https://www.lancaster.ac.uk/media/lancaster-university/content-assets/documents/fhm/dhr/chir/NSsynthesisguidanceVersion1-April2006.pdf
18. Cochrane Collaboration Training. Cochrane Handbook for Systematic Reviews of Interventions. 2022.
19. Ogilvie D, Fayter D, Petticrew M, et al. The harvest plot: a method for synthesising evidence about the differential effects of interventions. *BMC medical research methodology*. 02/25/2008 2008;8doi:10.1186/1471-2288-8-8
20. *RStudio*. 2020. **http://www.rstudio.com/**

For peer review only

Appendix 1 – Preliminary Search Strategy for MEDLINE

Ovid MEDLINE(R) ALL <1946 to December 09, 2022>

1 (Aruba* or Andorra* or "United Arab Emirates" or UAE or Emirati* or Antigua* or Barbuda* or Australia* or Austria* or Belgi* or Bahrain* or Baham* or Bermuda* or Barbad* or Brunei* or Canada* or Switzerland or Swiss or "Channel Island*" or Chile or Chilean* or Curacao* or "Cayman Island*" or Caymanian* or Cypr* or Czech* or German* or Denmark or Danish or Spain* or Estonia* or Finland or Finnish or France or French or Faroe* or "United Kingdom" or UK or England or Wales or welsh or Scotland or Brit* or Scot* or English or Gibraltar * or Gree* or Greenland* or Guam* or Chamorros or Hong Kong* or Croatia* or Hungar* or "Isle of Man" or Manx or Ireland or Irish or Iceland* or Israel* or Ital* or Japan* or "St Kitts" or Korea* or Kuwait* or Liechtenstein* or Lithuania* or Luxembourg* or Latvia* or Maca* or "St Martin*" or "Sint Maarten*" or Monaco or Monegasqu* or Malta or Maltese or "Northern Mariana Island*" or "New Caledonia*" or Netherlands or Dutch or Norw* or Nauru* or "New Zealand" or Kiwi or Oman* or Palau* or Poland or Polish or "Puerto Ric*" or Portug* or "French Polynesia*" or Qatar* or "Saudi Arabia*" or Singapor* or "San Marino" or "Sammarinese" or Slovak* or Slovenia* or Sweden or Swedish or Seychell* or Creole or "Turks Caicos" or Trinidad* or Tobago* or Uruguay* or "United States" or USA or America* or Venezuela* or "Virgin Island*" or EU or "European Union" or European*).ti,ab.3264235
2 developed countries/ or european union/38606
3 developed countr*.mp. 59651
4 (high income countr* or high-income countr*).mp. 11176
5 industrialised countr*.mp. 1502
6 more economically developed countr*.mp. 36
7 MEDC.mp. 43
8 advanced countr*.mp. 492
9 advanced econom*.mp. 233
10 developed econom*.mp. 455
11 industrialized econom*.mp. 43
12 industrialised econom*.mp. 11
13 (Migrant* or refugee* or immigrant* or foreigner* or newcomer* or new-comer* or migration or immigration or emigrant* or nonnative* or non-native* or diaspora or border crossing* or transient* or asylum-seek*).mp. 782544
14 ((displaced or undocumented or foreign* or resettle* or settle* or displace*) adj3 (person* or people)).mp. 4461
15 exp Human Migration/ 27663
16 Refugees/ 12718
17 "Transients and Migrants"/ 13851
18 13 or 14 or 15 or 16 or 17 784919
19 (pregnan* or postpartum or post-partum or postnatal or post-natal or antenatal or ante-natal or prenatal or pre-natal or antepartum or ante-partum or peripartum or peri-partum or birth* or trimester* or mother* or mum or mums or mom or moms or perinatal or peri-natal or childbirth* or obstetric* or puerper* or maternity or maternal).mp. 1856432
20 exp peripartum period/ or exp postpartum period/ or exp pregnancy/1013266
21 19 or 20 1878022

For peer review only - http://bmjopen.bmj.com/site/about/guidelines.xhtml

22	(intervention* or prevent* or program* or strateg* or class* or special*).mp.	7862429
23	(Peer-led or "peer led" or doula* or midwi*).mp.	41688
24	((antenatal or Postnatal) and (session* or lesson* or workshop*)).mp.	1994
25	22 or 23 or 24	7888583
26	1 and 18 and 21 and 25	4520
27	2 or 3 or 4 or 5 or 6 or 7 or 8 or 9 or 10 or 11 or 12	89366
28	18 and 21 and 27	1198
29	26 or 28	5360

For peer review only

PRISMA-P (Preferred Reporting Items for Systematic review and Meta-Analysis Protocols) 2015 checklist: recommended items to address in a systematic review protocol*

Section and topic	Item No	Checklist item
**ADMINISTRATIVE INFORMATION**		
Title:		
Identification	1a	Identify the report as a protocol of a systematic review
Update	1b	If the protocol is for an update of a previous systematic review, identify as such
Registration	2	If registered, provide the name of the registry (such as PROSPERO) and registration number
Authors:		
Contact	3a	Provide name, institutional affiliation, e-mail address of all protocol authors; provide physical mailing address of corresponding author
Contributions	3b	Describe contributions of protocol authors and identify the guarantor of the review
Amendments	4	If the protocol represents an amendment of a previously completed or published protocol, identify as such and list changes; otherwise, state plan for documenting important protocol amendments
Support:		
Sources	5a	Indicate sources of financial or other support for the review
Sponsor	5b	Provide name for the review funder and/or sponsor
Role of sponsor or funder	5c	Describe roles of funder(s), sponsor(s), and/or institution(s), if any, in developing the protocol
**INTRODUCTION**		
Rationale	6	Describe the rationale for the review in the context of what is already known
Objectives	7	Provide an explicit statement of the question(s) the review will address with reference to participants, interventions, comparators, and outcomes (PICO)
**METHODS**		
Eligibility criteria	8	Specify the study characteristics (such as PICO, study design, setting, time frame) and report characteristics (such as years considered, language, publication status) to be used as criteria for eligibility for the review
Information sources	9	Describe all intended information sources (such as electronic databases, contact with study authors, trial registers or other grey literature sources) with planned dates of coverage
Search strategy	10	Present draft of search strategy to be used for at least one electronic database, including planned limits, such that it could be repeated
Study records:		
Data management	11a	Describe the mechanism(s) that will be used to manage records and data throughout the review

For peer review only

Selection process	11b	State the process that will be used for selecting studies (such as two independent reviewers) through each phase of the review (that is, screening, eligibility and inclusion in meta-analysis)
Data collection process	11c	Describe planned method of extracting data from reports (such as piloting forms, done independently, in duplicate), any processes for obtaining and confirming data from investigators
Data items	12	List and define all variables for which data will be sought (such as PICO items, funding sources), any pre-planned data assumptions and simplifications
Outcomes and prioritization	13	List and define all outcomes for which data will be sought, including prioritization of main and additional outcomes, with rationale
Risk of bias in individual studies	14	Describe anticipated methods for assessing risk of bias of individual studies, including whether this will be done at the outcome or study level, or both; state how this information will be used in data synthesis
Data synthesis	15a	Describe criteria under which study data will be quantitatively synthesised
	15b	If data are appropriate for quantitative synthesis, describe planned summary measures, methods of handling data and methods of combining data from studies, including any planned exploration of consistency (such as I^2, Kendall's τ)
	15c	Describe any proposed additional analyses (such as sensitivity or subgroup analyses, meta-regression)
	15d	If quantitative synthesis is not appropriate, describe the type of summary planned
Meta-bias(es)	16	Specify any planned assessment of meta-bias(es) (such as publication bias across studies, selective reporting within studies)
Confidence in cumulative evidence	17	Describe how the strength of the body of evidence will be assessed (such as GRADE)

*** It is strongly recommended that this checklist be read in conjunction with the PRISMA-P Explanation and Elaboration (cite when available) for important clarification on the items. Amendments to a review protocol should be tracked and dated. The copyright for PRISMA-P (including checklist) is held by the PRISMA-P Group and is distributed under a Creative Commons Attribution Licence 4.0.**

From: Shamseer L, Moher D, Clarke M, Ghersi D, Liberati A, Petticrew M, Shekelle P, Stewart L, PRISMA-P Group. Preferred reporting items for systematic review and meta-analysis protocols (PRISMA-P) 2015: elaboration and explanation. BMJ. 2015 Jan 2;349(jan02 1):g7647.

For peer review only

BMJ Open

Interventions to Improve Perinatal Outcomes amongst Migrant Women in High-Income Countries: A Systematic Review Protocol

Journal:	*BMJ Open*
Manuscript ID	bmjopen-2023-072090.R1
Article Type:	Protocol
Date Submitted by the Author:	13-Jun-2023
Complete List of Authors:	Stevenson, Kerrie; University College London, Institute of Health Informatics<br>Ogunlana, K; University College London, Institute of Health Informatics<br>Edwards, Samuel; University of Warwick, Warwick Medical School<br>Henderson, William; University College London, Library Services<br>Rayment-Jones, Hannah; King's College London, Women and Children's Health<br>McGranahan, Majel; University of Warwick<br>Marti-Castaner, Maria; University of Copenhagen, Health Department of Public Health<br>Fellmeth, Gracia; University of Oxford Nuffield Department of Population Health<br>Luchenski, Serena; University College London, Institute of Health Informatics<br>Stevenson, Fiona; University College London, Primary Care and Population Sciences<br>Knight, Marian; University of Oxford, National Perinatal Epidemiology Unit<br>Aldridge, Robert; University College London, Institute of Health Informatics
**Primary Subject Heading**:	Obstetrics and gynaecology
Secondary Subject Heading:	Public health
Keywords:	OBSTETRICS, PUBLIC HEALTH, PAEDIATRICS

SCHOLARONE™
Manuscripts

Interventions to Improve Perinatal Outcomes amongst Migrant Women in High-Income Countries: A Systematic Review Protocol

Authors

K Stevenson[1*], K Ogunlana[1], S Edwards[2], W Henderson[3], H Raymont-Jones[4], M McGranahan[2], M Marti-Castaner[5], G Fellmeth[6], S Luchenski[7], F Stevenson[8], M Knight[5], R Aldridge[1]

Affiliations

1. Institute of Health Informatics, University College London, 222 Euston Road, London, NW1 2DA, UK.
2. Warwick Medical School, University of Warwick, Coventry, CV4 7HL, UK.
3. Library Services, University College London, Gower Street, London, WC1E 6BT, UK.
4. Department of Women and Children's Health, King's College London, Strand, London, WC2R 2LS, UK.
5. Danish Research Centre for Migration, Ethnicity, and Health, Department of Public Health, University of Copenhagen, Øster Farimagsgade 5,1014 København K, Denmark.
6. National Perinatal Epidemiology Unit, Nuffield Department of Population Health, University of Oxford, Old Road Campus, Headington, Oxford OX3 7LF, UK.
7. Collaborative Centre for Inclusion Health, University College London, 1-19 Torrington Place, London, WC1E 7HB UK.
8. Research Department of Primary Care and Health, Royal Free Campus, Rowland Hill Street, London, NW33 2PF, UK.

***Corresponding Author**

Dr Kerrie Stevenson

Email: k.stevenson@ucl.ac.uk

Address: Institute of Health Informatics, University College London, 222 Euston Road, London, NW1 2DA

For peer review only

ABSTRACT

Introduction

Women who are migrants and who are pregnant or postpartum are at high risk of poorer perinatal outcomes compared to host country populations due to experiencing numerous additional stressors including social exclusion and language barriers. High-income countries (HICs) host many migrants, including forced migrants who may face additional challenges in the peripartum period. Although HICs' maternity care systems are often well developed, they are not routinely tailored to the needs of migrant women. The primary objective of this systematic review is to provide an assessment of what interventions exist to improve perinatal outcomes in HICs. The secondary objective is to assess the most effective interventions for improving perinatal outcomes in HICs.

Methods and Analysis

This protocol follows the Preferred Reporting Items for Systematic Reviews and Meta-Analyses Protocols guidelines. EMBASE, EMCARE, MEDLINE and PsycInfo, CENTRAL, Scopus, CINAHL Plus, and Web of Science, as well as grey literature sources will be searched from inception up until December 2022. We will include randomised-controlled trials, quasi-experimental and interventional studies of interventions which aim to improve perinatal outcomes in any HIC. There will be no language restrictions. We will exclude studies presenting only qualitative outcomes and those including mixed populations of migrant and non-migrant women. Screening and data extraction will be completed by two independent reviewers and risk of bias will be assessed using the Quality Assessment Tool for Quantitative Studies. If a collection of suitably comparable outcomes is retrieved, we will perform meta-analysis applying a random effects model. Presentation of results will comply with guidelines in the Cochrane Handbook of Systematic Reviews of Interventions and the PRISMA statement.

Ethics and Dissemination

For peer review only

Ethical approval is not required. Results will be submitted for peer-reviewed publication and presented at national and international conferences. The findings will inform the work of the Lancet Migration European Hub.

PROSPERO Registration Number

CRD42022380678

Strengths and Limitations of this Study

- We will employ rigorous methodology in accordance with the Cochrane Handbook of Systematic Reviews and report in accordance with the Preferred Reporting Items for Systematic Reviews and Meta-Analyses Protocol (PRISMA-P) statement.
- We aim to assess if included studies employed co-production methods at any point in intervention development or analysis.
- The search strategy was developed with an experienced medical librarian and adapted for each database searched.
- No language restrictions will be employed, and we are doing an extensive and systematic grey literature search which is often omitted in similar reviews.
- The certainty of evidence may be limited by the number of studies available and because some studies may be of low quality without a quantitative outcome assessment.

For peer review only

INTRODUCTION

Rationale

There are an estimated 281 million international migrants in the world, constituting 3.6% of the global population.[1] Of these, approximately 13% are forced migrants, including refugees and asylum seekers who often face significant hardships during migration and when settled in their host country.[2] The remainder are economic migrants who often choose to migrate to reunite with family or for better job prospects, but they may also experience marginalisation due to poverty, social isolation, and discrimination.[1] Between 2000 and 2022 the international migrant population increased by 108 million, and although the majority of international migrants originate from low- and middle-income countries (LMICs), increasing numbers hope to settle in high-income countries (HICs).[1] HICs are not always well adapted to care for the needs of marginalised migrants, and many HICs struggle to adapt to this changing migration landscape.[1]

Women constitute approximately half of the international migrant population.[2] Women who migrate and who are pregnant or post-partum can face significant barriers to accessing maternity care, putting them at increased risk of poor mental and physical health.[3-6] They may experience poor social support in their host country, an inability to access to healthcare, and language barriers.[5,7] Forced migrant women who are pregnant or post-partum experience additional challenges including pre-migration stressors such as the trauma of war, transition stressors including dangerous migration journeys and gender-based violence, and post-migration stressors such as poor access to legal entitlements, discrimination, and socio-cultural barriers in obstetric care.[7,8] They are also more likely to have poor access to antenatal care and experience higher rates of perinatal mortality, miscarriage, and stillbirth than non-refugee women.[9] HICs often have some of the resources needed to support marginalised pregnant and postpartum migrant women, but often struggle to provide optimum care.[8]

For peer review only

To our knowledge no previous review has sought to quantitatively synthesise the literature on the most effective interventions to improve perinatal outcomes for migrant women in HICs. Balaam et al. conducted a systematic review in 2020 which aimed to identify social support interventions for asylum-seeking and refugee women in Europe.[10] The findings were qualitatively synthesised and women valued peer support and interventions that addressed their needs in a holistic way.[10] The UK National Institute of Health and Care Research (NIHR) commissioned a systematic review to explore interventions to improve maternity care for migrant women in the UK in 2017.[11] The review included only UK studies and identified just four interventions. These included peer support and specialist maternity care interventions, but they were not robustly evaluated so it was difficult to draw conclusions on their effectiveness.[11]

Objectives

This systematic review aims to identify the most effective interventions to improve perinatal outcomes for migrant women in HICs by quantitatively synthesising the literature. The primary objective will be to determine what interventions exist to improve perinatal outcomes for migrant women in HICs. The secondary objective will be to explore the effectiveness of these interventions by exploring the impact on perinatal outcomes. The main outcomes of interest will be rates of pre-term birth, birthweight, and number of antenatal or postnatal appointments attended.

METHODS AND ANALYSIS

Registration and Protocol Adherence

This systematic review will be reported in accordance with the Preferred Reporting Items for Systematic Reviews and Meta-Analyses (PRISMA) guidelines and was registered on PROSPERO: CRD42022380678 on 9th December 2022.

Definitions

For peer review only

For the purposes of this review, migrant women will be defined as being aged 16 years or older and who were born outside their host country. Eligible maternity care interventions are any hospital- or community-based initiatives offered in the antenatal, perinatal, or postnatal period up to one year postpartum. The perinatal period is defined as pregnancy and up to one year postpartum.

Eligibility Criteria and Patient, Intervention, Comparison Outcome (PICO) Framework

PICO Framework

- Population: Perinatal migrant women (those who were not born in their host country) aged 16 years or older and living in HICs (defined as being in the World Bank high-income economy category).[12]
- Intervention: Any hospital- or community-based activity undertaken with the aim of improving perinatal outcomes and delivered during the antenatal period and up to one year postpartum.
- Control: Usual care if data are available.
- Outcome: Improvements in perinatal outcomes (rates of miscarriage, preterm birth, stillbirth, birthweight, mode of delivery, APGAR score, maternal/neonatal critical care admission, breastfeeding initiation and duration, maternal/neonatal death, perinatal mental illness); number of antenatal or postnatal appointments attended; or change in maternal wellbeing as assessed by validated mental illness or wellbeing screening scales. The main outcomes of interest will be rates of pre-term birth, birthweight, and number of antenatal or postnatal appointments attended as these are key mediators of longer-term health of the infant and crucial measures of quality of maternity care in accordance with the World Health Organisation guidelines for Quality of Care for Pregnant Women and Newborns.[13]

Inclusion and Exclusion Criteria

For peer review only

Observational, quasi-experimental, and experimental intervention studies published from inception will be included. Abstracts, non-empirical research, opinion, or editorial pieces will be excluded. If duplicate reports or publications of the same data are retrieved, the less complete or recent version will be excluded if the same data are reported. Studies including only a qualitative outcome assessment will be excluded. Interventions that were not specifically designed or adapted for migrant women in the perinatal period will be excluded. This is to ensure our results are focused on interventions that could be directly implemented for migrant women and have direct impact their outcomes. Additionally, we felt this ensures the systematic review is focused and won't return an unmanageable number of results.

Patient and Public Involvement

Migrant women who are residing in the UK helped with the review's inception and design, and will also help with data extraction, analysis, and interpretation. They will also help with disseminating the work through co-authorship on peer-reviewed manuscripts and presentations at conferences.

Search Strategy and Data Sources

EMBASE, EMCARE, MEDLINE and PsycInfo via Ovid, Cochrane Central Register of Controlled Trials (CENTRAL) via Cochrane Library, Scopus, CINAHL Plus via EBSCOHost, and Web of Science from inception up until December 2022 (Appendix 1). Grey literature sources including Google Scholar and trial registries were searched up until December 2022. The first 150 results from the following supplementary sources will be searched: Google Scholar, WHO International Clinical Trials Registry Platform (ICTRP), Clinicaltrials.gov, the World Health Organisation Website, and the UN Refugee Agency website. Forward citation searching will be employed on all included articles, and the reference lists of all included articles will also be searched. No language restrictions will be employed. If studies are published in a language other than English, one of the research team who is fluent in that language will assess for likely relevance and extract the data, if

For peer review only

appropriate. The article will also be translated using Google Translate™ and a second reviewer will review its relevance and extract the data, if appropriate. If none of the research team are fluent in the language, we will pay UCL's graduate linguistics programme to translate article. The Boolean operators "AND" and "OR" will be employed to combine the descriptors. An experienced medical librarian helped to develop the search strategy and it has been adapted for each database. A search strategy is provided in Appendix 1. EndNote™ will be used to collect and manage studies retrieved.[14] Covidence™ will be used for deduplication and for study selection.[15]

Data Extraction (Selection and Coding)

Two independent reviewers will screen the titles and abstracts of all the citations for relevance. Full text manuscripts for relevant articles will be obtained. Full texts will be independently assessed for eligibility using a checklist of the inclusion and exclusion criteria by two independent reviewers. Studies meeting the inclusion criteria will be selected for inclusion in the review. All excluded articles from the full text screening will be retained with reason for exclusion noted. Disagreements between reviewers will be discussed and agreement sought from a third reviewer if necessary. If data are not accessible from the paper, the authors will be contacted. All extracted data will be recorded on the piloted data extraction form by two separate reviewers and cross-checked. The main data fields will be: (a) Author, publication year; (b) Country; (c) Study design; (d) Population and baseline characteristics; (e) Context (community or hospital-based or online); (f) Intervention details; (g) Control or comparison; (h) Timing of outcome measurements; (i) Outcome measures (type, scale used, scale validation status); (j) Outcomes; (k) Quality assessment; (l) Reported according to CONSORT guidelines; (m) Co-production methods used. A flowchart will summarise the selection process in line with the PRISMA 2020 guidelines. Study characteristics will be summarised and presented in tables.

Quality Assessment / Risk of Bias

For peer review only

Two reviewers will perform the critical appraisal independently and this will be independently cross-checked. The Quality Assessment Tool for Quantitative Studies will be used to assess rigor for each included study.[16] This was chosen as it has been developed and validated to assess both observational and experimental studies and shows reliability and validity. It assesses selection bias, study design, confounders, blinding, data collection, withdrawals, intervention integrity, and statistical analysis. If meta-analysis is conducted, publication bias will be assessed using a funnel plot.

Data Synthesis

Study characteristics including outcomes e.g., raw proportions, mean scores, and odds/risk ratios will be extracted and presented in tabular form. Narrative synthesis will be conducted according to Cochrane guidance and will include the creation of categories of interventions based on included papers e.g. specialist midwifery services, interpreting services, and use of a doula.[17,18] If appropriate, heterogeneity between studies will be explored using the I^2 statistic. Random effects meta-analysis will be conducted with 95% confidence intervals to allow for expected heterogeneity between different study populations, if appropriate. If possible, pooled estimates of odds/risk ratios with 95% confidence intervals will be calculated to explore outcomes among migrant women compared to usual care. Subgroup analyses according to migration status (economic and forced) and study context (hospital- or community-based) will be conducted, if appropriate. Sensitivity analysis according to study quality and method of recruitment will be undertaken. If meta-analysis is not possible due to a lack of standardised outcome data, alternative quantitative methods will be used following the Cochrane Handbook for Systematic Reviews of Interventions guidance.[18] The handbook details several approaches, but it is likely a Harvest Plot will be most appropriate. A Harvest Plot provides a visual extension of vote counting by categorising studies based on their effect (e.g. 'beneficial effect' or 'detrimental effect').[19] Vote counting is recommended when there are inconsistent effect measures across studies. Traditional vote counting methods using statistical significance, magnitude of effect, or subjective rules have been shown to be

For peer review only

misleading.[18] Instead, we will create a standardised binary metric, 'beneficial' or 'detrimental' based on direction of effect for each intervention according to outcome category (e.g. birthweight, stillbirth rates, or antenatal appointment attendance).[18] We will calculate the proportion of beneficial studies, 95% confidence interval (binomial exact calculation), and p-value (binomial probability test) to demonstrate if there is any evidence of an effect. We will present the findings on a Harvest Plot which will take the form of a 'supermatrix' which visually displays the vote counting results for each intervention.[18] All statistical analyses will be conducted using R Studio.[20] Meta-biases such as publication bias across studies will not be assessed.

Confidence in Cumulative Evidence

Confidence in the strength of evidence found will be assessed using the overall assessment outlined in the Quality Assessment Tool for Quantitative Studies checklist.[16] This will be done by two reviewers and possible disagreement will be assessed by third reviewer.

Ethics and Dissemination

Ethical approval is not required for this study as no primary data are being collected. We intend to publish the results in a peer-reviewed open access publication and present results at national and international conferences. The findings will inform the work of the Lancet Migration European Regional Hub.

DISCUSSION

This systematic review will provide a comprehensive overview of interventions being used to improve perinatal outcomes for migrant women in HICs. It will make a systematic assessment of the most effective interventions which will help to inform policy and clinical decision-making across these regions. The findings can also be used by researchers

For peer review only

planning or adapting maternity care interventions for migrant women in HICs, as well as to guide resource allocation decisions by funders and healthcare managers. Potential limitations include the retrieval of low- quality studies with poor evaluation of outcomes which will inhibit our ability to robustly assess the evidence of effectiveness of these interventions. Attempts to retrieve all available literature have been utilised such as involving a medical librarian in the development of our search strategy to ensure a broad and sensitive selection of search terms and searching multiple databases and additional supplementary and grey literature sources, however it is possible that some relevant studies/data may be missed. The certainty of evidence may also be limited by few studies including quantitative outcome assessments of interventions. Additionally, we are only including studies conducted in HICs which means we may miss effective interventions which were developed and tested in LMICs. Studies including antenatal interventions and subsequent perinatal outcomes can be affected by time-related biases if women are recruited at various points throughout their pregnancy, i.e. person-time of observation is not properly accounted for in the design or analysis of a study.[21] We are unable to adjust for these biases in quantitatively synthesising our findings, but will consider the implications of this in our conclusions. We are taking a co-production approach to the planning, conducting, and interpretation of this work by ensuring migrant women who have given birth or been pregnant in their host country are involved throughout. We will also be assessing if included studies have taken a co-production approach at any point in intervention development, implementation, or evaluation to gain an understanding of the prevalence of co-production in this field. We will identify gaps in the evidence to guide future research priorities.

For peer review only

Contributors

KS, KO, FS, MN, RA conceived of the review. KS, KO, SE, WH, HRJ, MM, MMC, GF, SL, FS, MN, RA developed the approach and protocol. KS developed the search strategy. KS

wrote the first draft of the protocol, and all authors were invited to comment. KS is the guarantor of the review. All authors approved the final version of the protocol.

Funding

KS is supported by a National Institute of Health and Care Research (NIHR) Doctoral Fellowship [Grant number NIHR302577]. GF is funded by a Clinical Research Fellowship from the Nuffield Department of Population Health, University of Oxford [Grant number N/A]. MM is supported by a Medical Research Council Clinical Research Training Fellowship [Grant number MR/W01498X/1]. The funders played no part in the design and writing of the protocol.

Competing Interests

None declared.

Word Count

2299.

References

1. UN Migration. *World Migration Report 2022*. 2022. https://www.iom.int/wmr/interactive
2. UN Refugee Agency. Figures at a glance. https://www.unhcr.org/uk/figures-at-a-glance.html
3. Sudbury H, Robinson A. Barriers to sexual and reproductive health care for refugee and asylum-seeking women. Article. *British Journal of Midwifery*. 2016;24(4):275-281. doi:10.12968/bjom.2016.24.4.275
4. Fair F, Raben L, Watson H, Vivilaki V, Muijsenbergh Mvd, Soltani H. Migrant women's experiences of pregnancy, childbirth and maternity care in European countries: a systematic review. *PLoS ONE*. 2020;15(2)doi:http://dx.doi.org/10.1371/journal.pone.0228378
5. Almeida LM, Caldas J, Ayres-De-Campos D, Salcedo-Barrientos D, Dias S. Maternal healthcare in migrants: A systematic review. Review. *Maternal and Child Health Journal*. 2013;17(8):1346-1354. doi:10.1007/s10995-012-1149-x
6. Anderson FM, Hatch SL, Comacchio C, Howard LM. Prevalence and risk of mental disorders in the perinatal period among migrant women: a systematic review

For peer review only

and meta-analysis. *Arch Womens Ment Health*. 2017;20(3):449-62. doi:10.1007/s00737-017-0723-z
7. Fair F, Watson H, Soltani H, Raben L, Vivilaki V, van den Muijsenbergh M. Migrant women's experiences of pregnancy, childbirth and maternity care in European countries: A systematic review. *PLoS ONE*. 2020;15(2):e0228378. doi:http://dx.doi.org/10.1371/journal.pone.0228378
8. World Health Organization Regional Office for Europe. *Improving the health care of pregnant refugee and migrant women and newborn children*. 2018. https://www.euro.who.int/__data/assets/pdf_file/0003/388362/tc-mother-eng.pdf
9. Sturrock S, Williams E, Greenough A. Antenatal and perinatal outcomes of refugees in high income countries. *Journal of perinatal medicine*. 2020;49(1):80-93. doi:https://dx.doi.org/10.1515/jpm-2020-0389
10. Balaam M-C, Kingdon C, Haith-Cooper M. A Systematic Review of Perinatal Social Support Interventions for Asylum-seeking and Refugee Women Residing in Europe. ReviewPaper. *Journal of Immigrant and Minority Health*. 2021-07-17 2021;24(3):741-758. doi:doi:10.1007/s10903-021-01242-3
11. Higginbottom GMA, Evans C, Hussain B, Morgan M, Bharj KK, Eldridge J. Experience of and access to maternity care in the UK by immigrant women: A narrative synthesis systematic review. *BMJ Open*. 2019;9(12):e029478. doi:http://dx.doi.org/10.1136/bmjopen-2019-029478
12. The World Bank. World Bank Country and Lending Groups. https://datahelpdesk.worldbank.org/knowledgebase/articles/906519
13. Tunçalp Ö, Were W, MacLennan C, et al. Quality of care for pregnant women and newborns-the WHO vision. *BJOG : an international journal of obstetrics and gynaecology*. 2015 Jul 2015;122(8)doi:10.1111/1471-0528.13451
14. *EndNote*. Version 20. Clarivate; 2013.
15. *Covidence Systematic Review Software*. Veritas Health Innovation; 2021.
16. Effective Public Healthcare Panacea Project. Quality Assessment Tool for Quantitative Studies. https://www.ephpp.ca/quality-assessment-tool-for-quantitative-studies/
17. Popay J, Roberts H, Sowden A, et al. *Guidance on the Conduct of Narrative Synthesis in Systematic Reviews*. 2006. https://www.lancaster.ac.uk/media/lancaster-university/content-assets/documents/fhm/dhr/chir/NSsynthesisguidanceVersion1-April2006.pdf
18. Cochrane Collaboration Training. Cochrane Handbook for Systematic Reviews of Interventions. 2022.
19. Ogilvie D, Fayter D, Petticrew M, et al. The harvest plot: a method for synthesising evidence about the differential effects of interventions. *BMC medical research methodology*. 02/25/2008 2008;8doi:10.1186/1471-2288-8-8
20. *RStudio*. 2020. **http://www.rstudio.com/**
21. Ukah U, Aibibula W, Platt R, Dayan N, Reynier P, Filion K. Time-related biases in perinatal pharmacoepidemiology: A systematic review of observational studies. *Pharmacoepidemiology and drug safety*. 2022 Dec 2022;31(12)doi:10.1002/pds.5504

For peer review only

APPENDIX 1 – Search Strategies

Emcare

1 (Aruba* or Andorra* or "United Arab Emirates" or UAE or Emirati* or Antigua* or Barbuda* or Australia* or Austria* or Belgi* or Bahrain* or Baham* or Bermuda* or Barbad* or Brunei* or Canada* or Switzerland or Swiss or "Channel Island*" or Chile or Chilean* or Curacao* or "Cayman Island*" or Caymanian* or Cypr* or Czech* or German* or Denmark or Danish or Spain* or Estonia* or Finland or Finnish or France or French or Faroe* or "United Kingdom" or UK or England or Wales or welsh or Scotland or Brit* or Scot* or English or Gibraltar * or Gree* or Greenland* or Guam* or Chamorros or Hong Kong* or Croatia* or Hungar* or "Isle of Man" or Manx or Ireland or Irish or Iceland* or Israel* or Ital* or Japan* or "St Kitts" or Korea* or Kuwait* or Liechtenstein* or Lithuania* or Luxembourg* or Latvia* or Maca* or "St Martin*" or "Sint Maarten*" or Monaco or Monegasqu* or Malta or Maltese or "Northern Mariana Island*" or "New Caledonia*" or Netherlands or Dutch or Norw* or Nauru* or "New Zealand" or Kiwi or Oman* or Palau* or Poland or Polish or "Puerto Ric*" or Portug* or "French Polynesia*" or Qatar* or "Saudi Arabia*" or Singapor* or "San Marino" or "Sammarinese" or Slovak* or Slovenia* or Sweden or Swedish or Seychell* or Creole or "Turks Caicos" or Trinidad* or Tobago* or Uruguay* or "United States" or USA or America* or Venezuela* or "Virgin Island*" or EU or "European Union" or European*).ti,ab. 1458038

2 developed country/ or european union/ 13481

3 developed countr*.mp. 13352

4 (high income countr* or high-income countr*).mp. 6622

5 industrialised countr*.mp. 623

6 more economically developed countr*.mp. 22

7 MEDC.mp. 8

8 advanced countr*.mp. 158

9 advanced econom*.mp. 92

10 developed econom*.mp. 161

11 industrialized econom*.mp. 11

12 industrialised econom*.mp. 8

13 (Migrant* or refugee* or immigrant* or foreigner* or newcomer* or new-comer* or migration or immigration or emigrant* or nonnative* or non-native* or diaspora or border crossing* or transient* or asylum-seek*).mp. 163314

14 ((displaced or undocumented or foreign* or resettle* or settle* or displace*) adj3 (person* or people)).mp. 1301

15 exp Migration/ 8976

16 exp Refugee/ 7217

17 13 or 14 or 15 or 16 163953

For peer review only

18 (pregnan* or postpartum or post-partum or postnatal or post-natal or puerper* or antenatal or ante-natal or prenatal or pre-natal or antepartum or ante-partum or peripartum or peri-partum or birth* or trimester* or mother* or mum or mums or mom or moms or perinatal or peri-natal or childbirth* or obstetric* or maternity or maternal).mp. 460639

19 exp perinatal period/ or exp puerperium/ or exp pregnancy/ 125861

20 18 or 19 464076

21 (intervention* or prevent* or program* or strateg* or class* or special*).mp. 2320749

22 (Peer-led or "peer led" or doula* or midwi*).mp. 22932

23 ((antenatal or Postnatal) and (session* or lesson* or workshop*)).mp. 1060

24 21 or 22 or 23 2333617

25 1 and 17 and 20 and 24 2274

26 2 or 3 or 4 or 5 or 6 or 7 or 8 or 9 or 10 or 11 or 12 27873

27 17 and 20 and 26 212

28 25 or 27 2410

Embase

1 (Aruba* or Andorra* or "United Arab Emirates" or UAE or Emirati* or Antigua* or Barbuda* or Australia* or Austria* or Belgi* or Bahrain* or Baham* or Bermuda* or Barbad* or Brunei* or Canada* or Switzerland or Swiss or "Channel Island*" or Chile or Chilean* or Curacao* or "Cayman Island*" or Caymanian* or Cypr* or Czech* or German* or Denmark or Danish or Spain* or Estonia* or Finland or Finnish or France or French or Faroe* or "United Kingdom" or UK or England or Wales or welsh or Scotland or Brit* or Scot* or English or Gibraltar * or Gree* or Greenland* or Guam* or Chamorros or Hong Kong* or Croatia* or Hungar* or "Isle of Man" or Manx or Ireland or Irish or Iceland* or Israel* or Ital* or Japan* or "St Kitts" or Korea* or Kuwait* or Liechtenstein* or Lithuania* or Luxembourg* or Latvia* or Maca* or "St Martin*" or "Sint Maarten*" or Monaco or Monegasqu* or Malta or Maltese or "Northern Mariana Island*" or "New Caledonia*" or Netherlands or Dutch or Norw* or Nauru* or "New Zealand" or Kiwi or Oman* or Palau* or Poland or Polish or "Puerto Ric*" or Portug* or "French Polynesia*" or Qatar* or "Saudi Arabia*" or Singapor* or "San Marino" or "Sammarinese" or Slovak* or Slovenia* or Sweden or Swedish or Seychell* or Creole or "Turks Caicos" or Trinidad* or Tobago* or Uruguay* or "United States" or USA or America* or Venezuela* or "Virgin Island*" or EU or "European Union" or European*).ti,ab. 5387298

2 developed country/ or european union/ 64842

3 developed countr*.mp. 65772

4 (high income countr* or high-income countr*).mp. 16400

5 industrialised countr*.mp. 1957

6 more economically developed countr*.mp. 49

7 MEDC.mp. 51

8 advanced countr*.mp. 668

9 advanced econom*.mp. 218

10 developed econom*.mp. 516

11 industrialized econom*.mp. 40

12 industrialised econom*.mp. 11

13 (Migrant* or refugee* or immigrant* or foreigner* or newcomer* or new-comer* or migration or immigration or emigrant* or nonnative* or non-native* or diaspora or border crossing* or transient* or asylum-seek*).mp. 1056344

14 ((displaced or undocumented or foreign* or resettle* or settle* or displace*) adj3 (person* or people)).mp. 2841

15 exp Migration/ 48713

16 exp Refugee/ 16436

17 13 or 14 or 15 or 16 1057673

18 (pregnan* or postpartum or post-partum or postnatal or post-natal or puerper* or antenatal or ante-natal or prenatal or pre-natal or antepartum or ante-partum or peripartum or peri-partum or birth* or trimester* or mother* or mum or mums or mom or moms or perinatal or peri-natal or childbirth* or obstetric* or maternity or maternal).mp. 2417880

19 exp perinatal period/ or exp puerperium/ or exp pregnancy/ 824664

20 18 or 19 2436673

21 (intervention* or prevent* or program* or strateg* or class* or special*).mp. 9841412

22 (Peer-led or "peer led" or doula* or midwi*).mp. 47440

23 ((antenatal or Postnatal) and (session* or lesson* or workshop*)).mp. 3201

24 21 or 22 or 23 9869943

25 1 and 17 and 20 and 24 6620

26 2 or 3 or 4 or 5 or 6 or 7 or 8 or 9 or 10 or 11 or 12 114481

27 17 and 20 and 26 956

28 25 or 27 7319

SCOPUS

TITLE-ABS-KEY (aruba* OR andorra* OR "United Arab Emirates" OR uae OR emirati* OR antigua* OR barbuda* OR australia* OR austria* OR belgi* OR bahrain* OR baham* OR bermuda* OR barbad* OR brunei* OR canada* OR switzerland OR swiss OR "Channel Islands" OR chile OR chilean* OR curacao* OR "Cayman Islands" OR caymanian* OR cypr* OR czech* OR german* OR denmark OR danish OR spain* OR estonia* OR finland OR finish OR france OR french OR faroe* OR

For peer review only

"United Kingdom" OR uk OR england OR wales OR scotland OR brit* OR scot* OR english OR "Gibraltar *" OR gree* OR greenland* OR guam* OR chamorros OR "Hong Kong*" OR croatia* OR hungar* OR "Isle of Man" OR manx OR ireland OR irish OR iceland* OR israel* OR ital* OR japan* OR "St Kitts" OR korea* OR kuwait* OR liechtenstein* OR lithuania* OR luxembourg* OR latvia* OR maca* OR "St Martin*" OR "Sint Maarten*" OR monaco OR monegasqu* OR malta OR maltese OR "Northern Mariana Island*" OR "New Caledonia*" OR netherlands OR dutch OR norw* OR nauru* OR "New Zealand" OR kiwi OR oman* OR palau* OR poland OR polish OR "Puerto Ric*" OR portug* OR "French Polynesia*" OR qatar* OR "Saudi Arabia*" OR singapor* OR "San Marino" OR sammarinese OR slovak* OR slovenia* OR sweden OR swedish OR seychell* OR creole OR "Turks Caicos" OR trinidad* OR tobago* OR uruguay* OR "United States" OR usa OR america* OR venezuela* OR "Virgin Island*" OR eu OR "European Union" OR european* OR "high income countr*" OR "high-income countr*" OR "industrialised countr*" OR "more economically developed countr*" OR "advanced countr*" OR "advanced econom*" OR "MEDC" OR "developed econom*" OR "industrialized econom*" OR "industrialised econom*") **AND** TITLE-ABS-KEY (migrant* OR refugee* OR immigrant* OR foreigner* OR newcomer* OR new-comer* OR migration OR immigration OR emigrant* OR nonnative* OR non-native* OR diaspora OR "border crossing*" OR transient* OR asylum-seek* OR "Displaced people*" OR "undocumented people*" OR "foreign* people*" OR "resettle* people*" OR "settle* people*" OR "Displaced person*" OR "undocumented person*" OR "foreign* person*" OR "resettle* person*" OR "settle* person*") **AND** TITLE-ABS-KEY (pregnan* OR postpartum OR post-partum OR postnatal OR post-natal OR puerperal OR antenatal OR ante-natal OR prenatal OR pre-natal OR antepartum OR ante-partum OR peripartum OR peri-partum OR birth* OR trimester* OR mother* OR mum OR mums OR mom OR moms OR perinatal OR peri-natal OR childbirth* OR obstetric* OR puerper* OR maternity OR maternal) **AND** TITLE-ABS-KEY ((intervention* OR prevent* OR program* OR strateg* OR class* OR special* OR peer-led OR "peer led" OR doula* OR midwi*) OR ((antenatal OR postnatal) AND (class* OR session* OR lesson* OR workshop*)))

8815

For peer review only

MEDLINE

Ovid MEDLINE(R) ALL <1946 to December 09, 2022>

1 (Aruba* or Andorra* or "United Arab Emirates" or UAE or Emirati* or Antigua* or Barbuda* or Australia* or Austria* or Belgi* or Bahrain* or Baham* or Bermuda* or Barbad* or Brunei* or Canada* or Switzerland or Swiss or "Channel Island*" or Chile or Chilean* or Curacao* or "Cayman Island*" or Caymanian* or Cypr* or Czech* or German* or Denmark or Danish or Spain* or Estonia* or Finland or Finnish or France or French or Faroe* or "United Kingdom" or UK or England or Wales or welsh or Scotland or Brit* or Scot* or English or Gibraltar * or Gree* or Greenland* or Guam* or Chamorros or Hong Kong* or Croatia* or Hungar* or "Isle of Man" or Manx or Ireland or Irish or Iceland* or Israel* or Ital* or Japan* or "St Kitts" or Korea* or Kuwait* or Liechtenstein* or Lithuania* or Luxembourg* or Latvia* or Maca* or "St Martin*" or "Sint Maarten*" or Monaco or Monegasqu* or Malta or Maltese or "Northern Mariana Island*" or "New Caledonia*" or Netherlands or Dutch or Norw* or Nauru* or "New Zealand" or Kiwi or Oman* or Palau* or Poland or Polish or "Puerto Ric*" or Portug*

or "French Polynesia*" or Qatar* or "Saudi Arabia*" or Singapor* or "San Marino" or "Sammarinese" or Slovak* or Slovenia* or Sweden or Swedish or Seychell* or Creole or "Turks Caicos" or Trinidad* or Tobago* or Uruguay* or "United States" or USA or America* or Venezuela* or "Virgin Island*" or EU or "European Union" or European*).ti,ab. 3264235

2 developed countries/ or european union/ 38606

3 developed countr*.mp. 59651

4 (high income countr* or high-income countr*).mp. 11176

5 industrialised countr*.mp. 1502

6 more economically developed countr*.mp. 36

7 MEDC.mp. 43

8 advanced countr*.mp. 492

9 advanced econom*.mp. 233

10 developed econom*.mp. 455

11 industrialized econom*.mp. 43

12 industrialised econom*.mp. 11

13 (Migrant* or refugee* or immigrant* or foreigner* or newcomer* or new-comer* or migration or immigration or emigrant* or nonnative* or non-native* or diaspora or border crossing* or transient* or asylum-seek*).mp. 782544

14 ((displaced or undocumented or foreign* or resettle* or settle* or displace*) adj3 (person* or people)).mp. 4461

15 exp Human Migration/ 27663

16 Refugees/ 12718

17 "Transients and Migrants"/ 13851

18 13 or 14 or 15 or 16 or 17 784919

19 (pregnan* or postpartum or post-partum or postnatal or post-natal or antenatal or ante-natal or prenatal or pre-natal or antepartum or ante-partum or peripartum or peri-partum or birth* or trimester* or mother* or mum or mums or mom or moms or perinatal or peri-natal or childbirth* or obstetric* or puerper* or maternity or maternal).mp. 1856432

20 exp peripartum period/ or exp postpartum period/ or exp pregnancy/ 1013266

21 19 or 20 1878022

22 (intervention* or prevent* or program* or strateg* or class* or special*).mp. 7862429

23 (Peer-led or "peer led" or doula* or midwi*).mp. 41688

24 ((antenatal or Postnatal) and (session* or lesson* or workshop*)).mp. 1994

25 22 or 23 or 24 7888583

26 1 and 18 and 21 and 25 4520

For peer review only

27	2 or 3 or 4 or 5 or 6 or 7 or 8 or 9 or 10 or 11 or 12	89366
28	18 and 21 and 27	1198
29	26 or 28	5360

Web of Science Search Strategy

Database: Web of Science Core Collection

Entitlements:

- WOS.IC: 1993 to 2022
- WOS.CCR: 1985 to 2022
- WOS.SCI: 1900 to 2022
- WOS.AHCI: 1975 to 2022
- WOS.BHCI: 2005 to 2022
- WOS.BSCI: 2005 to 2022
- WOS.ESCI: 2015 to 2022
- WOS.ISTP: 1990 to 2022
- WOS.SSCI: 1900 to 2022
- WOS.ISSHP: 1990 to 2022

Searches:

1: TS=(Aruba* OR Andorra* OR "United Arab Emirates" OR UAE OR Emirati* OR Antigua* OR Barbuda* OR Australia* OR Austria* OR Belgi* OR Bahrain* OR Baham* OR Bermuda* OR Barbad* OR Brunei* OR Canada* OR Switzerland OR Swiss OR "Channel Island*" OR Chile OR Chilean* OR Curacao* OR "Cayman Island*" OR Caymanian* OR Cypr* OR Czech* OR German* OR Denmark OR Danish OR Spain* OR Estonia* OR Finland OR Finnish OR France OR French OR Faroe* OR "United Kingdom" OR UK OR England OR Wales OR welsh OR Scotland OR Brit* OR Scot* OR English OR "Gibraltar *" OR Gree* OR Greenland* OR Guam* OR Chamorros OR "Hong Kong*" OR Croatia* OR Hungar* OR "Isle of Man" OR Manx OR Ireland OR Irish OR Iceland* OR Israel* OR Ital* OR Japan* OR "St Kitts" OR Korea* OR Kuwait* OR Liechtenstein* OR Lithuania* OR Luxembourg* OR Latvia* OR Maca* OR "St Martin*" OR "Sint Maarten*" OR Monaco OR Monegasqu* OR Malta OR Maltese OR "Northern Mariana Island*" OR "New Caledonia*" OR Netherlands OR Dutch OR Norw* OR Nauru* OR "New Zealand" OR Kiwi OR Oman* OR Palau* OR Poland OR Polish OR "Puerto Ric*" OR Portug* OR "French Polynesia*" OR Qatar* OR "Saudi Arabia*"

For peer review only

OR Singapor* OR "San Marino" OR Sammarinese OR Slovak* OR Slovenia* OR Sweden OR Swedish OR Seychell* OR Creole OR "Turks Caicos" OR Trinidad* OR Tobago* OR Uruguay* OR "United States" OR USA OR America* OR Venezuela* OR "Virgin Island*" OR EU OR "European Union" OR European* OR "high income countr*" OR "high-income countr*" OR "industrialised countr*" OR "more economically developed countr*" OR "advanced countr*" OR "advanced econom*" OR "MEDC" OR "developed econom*" OR "industrialized econom*" OR "industrialised econom*")

Date Run: Mon Dec 12 2022 13:10:27 GMT+0000 (Greenwich Mean Time)
Results: 10269737

2: TS=(migrant* OR refugee* OR immigrant* OR foreigner* OR newcomer* OR new-comer* OR migration OR immigration OR emigrant* OR nonnative* OR non-native* OR diaspora OR "border crossing*" OR transient* OR asylum-seek* OR "Displaced people*" OR "undocumented people*" OR "foreign* people*" OR "resettle* people*" OR "settle* people*" OR "Displaced person*" OR "undocumented person*" OR "foreign* person*" OR "resettle* person*" OR "settle* person*")

Date Run: Mon Dec 12 2022 13:12:33 GMT+0000 (Greenwich Mean Time)
Results: 1534587

3: TS=(pregnan* OR postpartum OR post-partum OR postnatal OR post-natal OR antenatal OR ante-natal OR prenatal OR pre-natal OR antepartum OR ante-partum OR peripartum OR peri-partum OR birth* OR trimester* OR mother* OR mum OR mums OR mom OR moms OR perinatal OR peri-natal OR childbirth* OR obstetric* OR puerper* OR maternity OR maternal)

Date Run: Mon Dec 12 2022 13:13:05 GMT+0000 (Greenwich Mean Time)
Results: 1676056

4: TS=((intervention* OR prevent* OR program* OR strateg* OR class* OR special* OR peer-led OR "peer led" OR doula* OR midwi*) OR ((antenatal OR postnatal) AND (class* OR session* OR lesson* OR workshop*)))

Date Run: Mon Dec 12 2022 13:13:46 GMT+0000 (Greenwich Mean Time)
Results: 12259143

5: #4 AND #3 AND #2 AND #1

Date Run: Mon Dec 12 2022 13:13:54 GMT+0000 (Greenwich Mean Time)
Results: 5662

For peer review only

APA PsycInfo <1806 to December Week 1 2022>

1 (Aruba* or Andorra* or "United Arab Emirates" or UAE or Emirati* or Antigua* or Barbuda* or Australia* or Austria* or Belgi* or Bahrain* or Baham* or Bermuda* or Barbad* or Brunei* or Canada* or Switzerland or Swiss or "Channel Island*" or Chile or Chilean* or Curacao* or "Cayman Island*" or Caymanian* or Cypr* or Czech* or German* or Denmark or Danish or Spain* or Estonia* or Finland or Finnish or France or French or Faroe* or "United Kingdom" or UK or England or Wales or welsh or Scotland or Brit* or Scot* or English or Gibraltar * or Gree* or Greenland* or Guam* or Chamorros or Hong Kong* or Croatia* or Hungar* or "Isle of Man" or Manx or Ireland or Irish or Iceland* or Israel* or Ital* or Japan* or "St Kitts" or Korea* or Kuwait* or Liechtenstein* or Lithuania* or Luxembourg* or Latvia* or Maca* or "St Martin*" or "Sint Maarten*" or Monaco or Monegasqu* or Malta or Maltese or "Northern Mariana Island*" or "New Caledonia*" or Netherlands or Dutch or Norw* or Nauru* or "New Zealand" or Kiwi or Oman* or Palau* or Poland or Polish or "Puerto Ric*" or Portug* or "French Polynesia*" or Qatar* or "Saudi Arabia*" or Singapor* or "San Marino" or "Sammarinese" or Slovak* or Slovenia* or Sweden or Swedish or Seychell* or Creole or "Turks Caicos" or Trinidad* or Tobago* or Uruguay* or "United States" or USA or America* or Venezuela* or "Virgin Island*" or EU or "European Union" or European*).ti,ab. 998036

2 developed countries/ 1444

3 developed countr*.mp. 6366

4 (high income countr* or high-income countr*).mp. 2276

5 industrialised countr*.mp. 162

6 more economically developed countr*.mp. 9

7 MEDC.mp. 4

8 advanced countr*.mp. 149

9 advanced econom*.mp. 164

10 developed econom*.mp. 377

11 industrialized econom*.mp. 54

12 industrialised econom*.mp. 3

13 (Migrant* or refugee* or immigrant* or foreigner* or newcomer* or new-comer* or migration or immigration or emigrant* or nonnative* or non-native* or diaspora or border crossing* or transient* or asylum-seek*).mp. 106941

14 ((displaced or undocumented or foreign* or resettle* or settle* or displace*) adj3 (person* or people)).mp. 1523

15 exp Human Migration/ 17211

16 13 or 14 or 15 108706

17 (pregnan* or postpartum or post-partum or postnatal or post-natal or antenatal or ante-natal or prenatal or pre-natal or antepartum or ante-partum or peripartum or peri-partum or birth* or trimester* or mother* or mum or mums or mom or moms or perinatal or peri-natal or childbirth* or obstetric* or puerper* or maternity or maternal).mp. 286207

For peer review only

18 exp Intrapartum Period/ or exp Antepartum Period/ or Perinatal Period/ or exp pregnancy/ or Postnatal Period/ or exp Birth/ or exp Prenatal Care/ 54584

19 17 or 18 287131

20 (intervention* or prevent* or program* or strateg* or class* or special*).mp. 1754113

21 (Peer-led or "peer led" or doula* or midwi*).mp. 5292

22 ((antenatal or Postnatal) and (session* or lesson* or workshop*)).mp. 857

23 20 or 21 or 22 1756811

24 1 and 16 and 19 and 23 1693

25 2 or 3 or 4 or 5 or 6 or 7 or 8 or 9 or 10 or 11 or 12 9141

26 16 and 19 and 25 69

27 24 or 26 1739

Cochrane Central Register of Controlled Trials

Issue 11 of 12, November 2022

Search Name: CL

Date Run: 22/12/2022 10:48:39

Comment:

ID	Search	Hits
#1	MeSH descriptor: [Developed Countries] explode all trees	52
#2	MeSH descriptor: [European Union] explode all trees	65
#3	(developed countr*):ti,ab,kw	4180
#4	(high income countr*):ti,ab,kw	2009
#5	(high-income countr*):ti,ab,kw	854
#6	(industrialised countr*):ti,ab,kw	459
#7	(economically developed countr*):ti,ab,kw	35
#8	(MEDC):ti,ab,kw	2
#9	(advanced countr*):ti,ab,kw	1500
#10	(advanced econom*):ti,ab,kw	957
#11	(developed econom*):ti,ab,kw	3125
#12	(industrialized econom*):ti,ab,kw	62

For peer review only

#13 (industrialised econom*):ti,ab,kw 62

#14 {OR #1-#13} 10948

#15 (Migrant*):ti,ab,kw 441

#16 (refugee):ti,ab,kw 444

#17 (immigrant*):ti,ab,kw 839

#18 (foreigner):ti,ab,kw 21

#19 (newcomer*):ti,ab,kw 48

#20 (new-comer*):ti,ab,kw 5

#21 (migration):ti,ab,kw 3759

#22 (immigration):ti,ab,kw 231

#23 (emigrant*):ti,ab,kw 238

#24 (nonnative*):ti,ab,kw 86

#25 (non-native*):ti,ab,kw 77

#26 (diaspora):ti,ab,kw 4

#27 (border crossing*):ti,ab,kw 16

#28 (transient*):ti,ab,kw 19243

#29 (asylum-seek*):ti,ab,kw 82

#30 ((displaced or undocumented or foreign* or resettle* or settle* or displace*) NEAR/3 (person* or people)):ti,ab,kw 89

#31 MeSH descriptor: [Human Migration] explode all trees 68

#32 MeSH descriptor: [Refugees] this term only 173

#33 MeSH descriptor: [Transients and Migrants] this term only 80

#34 {OR #15-#33} 24729

#35 (pregnan* or postpartum or post-partum or postnatal or post-natal or antenatal or ante-natal or prenatal or pre-natal or antepartum or ante-partum or peripartum or peri-partum or birth* or trimester* or mother* or mum or mums or mom or moms or perinatal or peri-natal or childbirth* or obstetric* or puerper* or maternity or maternal):ti,ab,kw 119742

#36 MeSH descriptor: [Peripartum Period] explode all trees 21

#37 MeSH descriptor: [Postpartum Period] explode all trees 1942

#38 MeSH descriptor: [Pregnancy] explode all trees 25029

#39 {OR #35-#38} 119890

#40 #14 AND #34 AND #39 54

For peer review only

Interface - EBSCOhost Research Databases
Search Screen - Advanced Search
Database - CINAHL Plus

#	Query	Limiters/Expanders	Results
S1	MH "developed countries"	Search modes - Boolean/Phrase	4,144
S2	(MH "European Union")	Search modes - Boolean/Phrase	6,324
S3	TX developed countr*	Search modes - Boolean/Phrase	14,015
S4	TX high income countr*	Search modes - Boolean/Phrase	5,938
S5	TX high-income countr*	Search modes - Boolean/Phrase	5,253
S6	TX industrialised countr*	Search modes - Boolean/Phrase	409
S7	TX economically developed countr*	Search modes - Boolean/Phrase	113
S8	TX MEDC	Search modes - Boolean/Phrase	9
S9	TX advanced countr*	Search modes - Boolean/Phrase	564
S10	TX advanced econom*	Search modes - Boolean/Phrase	406
S11	TX developed econom*	Search modes - Boolean/Phrase	1,039
S12	TX industrialized econom*	Search modes - Boolean/Phrase	50
S13	TX industrialised econom*	Search modes - Boolean/Phrase	11
S14	S1 OR S2 OR S3 OR S4 OR S5 OR S6 OR S7 OR S8 OR S9 OR S10 OR S11 OR S12 OR S13	Search modes - Boolean/Phrase	27,284
S15	TX Migrant* or refugee* or immigrant* or foreigner* or	Search modes - Boolean/Phrase	106,229

For peer review only

	newcomer* or new-comer* or migration or immigration or emigrant* or nonnative* or non-native* or diaspora or border crossing* or transient* or asylum-seek*		
S16	TX (displaced or undocumented or foreign* or resettle* or settle* or displace*) n3 (person* or people)	Search modes - Boolean/Phrase	1,998
S17	(MH "Refugees+") OR (MH "Transients and Migrants")	Search modes - Boolean/Phrase	14,390
S18	S15 OR S16 OR S17	Search modes - Boolean/Phrase	107,366
S19	TX pregnan* or postpartum or post-partum or postnatal or post-natal or antenatal or ante-natal or prenatal or pre-natal or antepartum or ante-partum or peripartum or peri-partum or birth* or trimester* or mother* or mum or mums or mom or moms or perinatal or peri-natal or childbirth* or obstetric* or puerper* or maternity or maternal	Search modes - Boolean/Phrase	502,585
S20	(MH "Pregnancy+")	Expanders - Apply equivalent subjects Search modes - Boolean/Phrase	246,130
S21	(MH "Postnatal Period+")	Expanders - Apply equivalent subjects Search modes - Boolean/Phrase	16,885

For peer review only

S22	(MH "Postnatal care+") OR (MH "Prenatal Care") OR (MH "Perinatal Care")	Search modes - Boolean/Phrase	29,500
S23	S19 OR S20 OR S21 OR S22	Search modes - Boolean/Phrase	504,522
S24	S14 AND S18 AND S23	Search modes - Boolean/Phrase	237
S25	TI (Aruba* or Andorra* or "United Arab Emirates" or UAE or Emirati* or Antigua* or Barbuda* or Australia* or Austria* or Belgi* or Bahrain* or Baham* or Bermuda* or Barbad* or Brunei* or Canada* or Switzerland or Swiss or "Channel Island*" or Chile or Chilean* or Curacao* or "Cayman Island*" or Caymanian* or Cypr* or Czech* or German* or Denmark or Danish or Spain* or Estonia* or Finland or Finnish or France or French or Faroe* or "United Kingdom" or UK or England or Wales or welsh or Scotland or Brit* or Scot* or English or Gibraltar * or Gree* or Greenland* or Guam* or Chamorros or Hong Kong* or Croatia* or Hungar* or "Isle of Man" or Manx or Ireland or Irish or Iceland* or Israel* or Ital* or Japan* or "St Kitts" or Korea* or Kuwait* or Liechtenstein* or	Search modes - Boolean/Phrase	1,194,855

For peer review only

	Lithuania* or Luxembourg* or Latvia* or Maca* or "St Martin*" or "Sint Maarten*" or Monaco or Monegasqu* or Malta or Maltese or "Northern Mariana Island*" or "New Caledonia*" or Netherlands or Dutch or Norw* or Nauru* or "New Zealand" or Kiwi or Oman* or Palau* or Poland or Polish or "Puerto Ric*" or Portug* or "French Polynesia*" or Qatar* or "Saudi Arabia*" or Singapor* or "San Marino" or "Sammarinese" or Slovak* or Slovenia* or Sweden or Swedish or Seychell* or Creole or "Turks Caicos" or Trinidad* or Tobago* or Uruguay* or "United States" or USA or America* or Venezuela* or "Virgin Island*" or EU or "European Union" or European*) OR AB (Aruba* or Andorra* or "United Arab Emirates" or UAE or Emirati* or Antigua* or Barbuda* or Australia* or Austria* or Belgi* or Bahrain* or Baham* or Bermuda* or Barbad* or Brunei* or Canada* or Switzerland or Swiss or "Channel Island*" or Chile or Chilean* or Curacao* or		

For peer review only

	"Cayman Island*" or Caymanian* or Cypr* or Czech* or German* or Denmark or Danish or Spain* or Estonia* or Finland or Finnish or France or French or Faroe* or "United Kingdom" or UK or England or Wales or welsh or Scotland or Brit* or Scot* or English or Gibraltar * or Gree* or Greenland* or Guam* or Chamorros or Hong Kong* or Croatia* or Hungar* or "Isle of Man" or Manx or Ireland or Irish or Iceland* or Israel* or Ital* or Japan* or "St Kitts" or Korea* or Kuwait* or Liechtenstein* or Lithuania* or Luxembourg* or Latvia* or Maca* or "St Martin*" or "Sint Maarten*" or Monaco or Monegasqu* or Malta or Maltese or "Northern Mariana Island*" or "New Caledonia*" or Netherlands or Dutch or Norw* or Nauru* or "New Zealand" or Kiwi or Oman* or Palau* or Poland or Polish or "Puerto Ric*" or Portug* or "French Polynesia*" or Qatar* or "Saudi Arabia*" or Singapor* or "San Marino" or "Sammarinese" or Slovak* or Slovenia* or Sweden or		

For peer review only

	Swedish or Seychell* or Creole or "Turks Caicos" or Trinidad* or Tobago* or Uruguay* or "United States" or USA or America* or Venezuela* or "Virgin Island*" or EU or "European Union" or European*)		
S26	S18 AND S23 AND S25	Search modes - Boolean/Phrase	4,466
S27	TX intervention* or prevent* or program* or strateg* or "classes" or "specialist" OR "specialists" OR Peer-led or "peer led" or doula* or midwi*	Search modes - Boolean/Phrase	2,112,926
S28	TX (antenatal or Postnatal) AND (session* or lesson* or workshop*)	Search modes - Boolean/Phrase	1,184
S29	S27 OR S28	Search modes - Boolean/Phrase	2,113,168
S30	S26 AND S29	Search modes - Boolean/Phrase	1,960
S31	S24 OR S30	Search modes - Boolean/Phrase	2,128

Grey Literature and Registry Searching:

WHO International Clinical Trials Registry Platform (ICTRP):

For peer review only

https://trialsearch.who.int/AdvSearch.aspx using Pregnancy AND migrant

Clinicaltrials.gov:

https://clinicaltrials.gov/ct2/search/advanced?cond=&term=&cntry=&state=&city=&dist= using the terms ‘Pregnancy’ ‘Migrant’ in the 'other terms' search field

Google Scholar:

Pregnancy refugee OR migrant OR asylum "high income"

World Health Organization Website:

site:who.int pregnancy refugee OR migrant OR asylum "high income": https://www.google.com/search?q=site%3Awho.int+pregnancy+refugee+OR+migrant+OR+asylum+%22high+income%22

UN Refugee Agency Website:

site:unhcr.org pregnancy refugee OR migrant OR asylum "high income": https://www.google.com/search?q=site%3Aunhcr.org+pregnancy+refugee+OR+migrant+OR+asylum+%22high+income%22

For peer review only

PRISMA-P (Preferred Reporting Items for Systematic review and Meta-Analysis Protocols) 2015 checklist: recommended items to address in a systematic review protocol*

Relevant Page number in 'red'.

Section and topic	Item No	Checklist item
**ADMINISTRATIVE INFORMATION**		
Title:		
Identification	1a	Identify the report as a protocol of a systematic review **Page 1**
Update	1b	If the protocol is for an update of a previous systematic review, identify as such **N/A**
Registration	2	If registered, provide the name of the registry (such as PROSPERO) and registration number **Page 3 & 5**
Authors:		
Contact	3a	Provide name, institutional affiliation, e-mail address of all protocol authors; provide physical mailing address of corresponding author **Page 1**
Contributions	3b	Describe contributions of protocol authors and identify the guarantor of the review **Page 12**
Amendments	4	If the protocol represents an amendment of a previously completed or published protocol, identify as such and list changes; otherwise, state plan for documenting important protocol amendments **N/A**
Support:		
Sources	5a	Indicate sources of financial or other support for the review **Page 12**
Sponsor	5b	Provide name for the review funder and/or sponsor **Page 12**
Role of sponsor or funder	5c	Describe roles of funder(s), sponsor(s), and/or institution(s), if any, in developing the protocol **Page 12**
**INTRODUCTION**		
Rationale	6	Describe the rationale for the review in the context of what is already known **Page 4**
Objectives	7	Provide an explicit statement of the question(s) the review will address with reference to participants, interventions, comparators, and outcomes (PICO) **Page 6**
**METHODS**		

For peer review only

Eligibility criteria	8	Specify the study characteristics (such as PICO, study design, setting, time frame) and report characteristics (such as years considered, language, publication status) to be used as criteria for eligibility for the review **Page 6 & 7**
Information sources	9	Describe all intended information sources (such as electronic databases, contact with study authors, trial registers or other grey literature sources) with planned dates of coverage **Page 7 & 8**
Search strategy	10	Present draft of search strategy to be used for at least one electronic database, including planned limits, such that it could be repeated **Appendix 1**
Study records: Data management	11a	Describe the mechanism(s) that will be used to manage records and data throughout the review **Page 7 & 8**
Selection process	11b	State the process that will be used for selecting studies (such as two independent reviewers) through each phase of the review (that is, screening, eligibility and inclusion in meta-analysis) **Page 7 & 8**
Data collection process	11c	Describe planned method of extracting data from reports (such as piloting forms, done independently, in duplicate), any processes for obtaining and confirming data from investigators **Page 7 & 8**
Data items	12	List and define all variables for which data will be sought (such as PICO items, funding sources), any pre-planned data assumptions and simplifications **Page 8**
Outcomes and prioritization	13	List and define all outcomes for which data will be sought, including prioritization of main and additional outcomes, with rationale **Page 6**
Risk of bias in individual studies	14	Describe anticipated methods for assessing risk of bias of individual studies, including whether this will be done at the outcome or study level, or both; state how this information will be used in data synthesis **Page 8 & 9**
Data synthesis	15a	Describe criteria under which study data will be quantitatively synthesised **Page 9**
	15b	If data are appropriate for quantitative synthesis, describe planned summary measures, methods of handling data and methods of combining data from studies, including any planned exploration of consistency (such as I^2, Kendall's τ) **Page 9 & 10**
	15c	Describe any proposed additional analyses (such as sensitivity or subgroup analyses, meta-regression) **Page 9 & 10**
	15d	If quantitative synthesis is not appropriate, describe the type of summary planned **Page 9 & 10**
Meta-bias(es)	16	Specify any planned assessment of meta-bias(es) (such as publication bias across studies, selective reporting within studies) **Page 10**
Confidence in cumulative evidence	17	Describe how the strength of the body of evidence will be assessed (such as GRADE) **Page 10**

*** It is strongly recommended that this checklist be read in conjunction with the PRISMA-P Explanation and Elaboration (cite when available) for important clarification on the items. Amendments to a review protocol should be tracked and dated. The copyright for PRISMA-P (including checklist) is held by the PRISMA-P Group and is distributed under a Creative Commons Attribution Licence 4.0.**

For peer review only - http://bmjopen.bmj.com/site/about/guidelines.xhtml

From: Shamseer L, Moher D, Clarke M, Ghersi D, Liberati A, Petticrew M, Shekelle P, Stewart L, PRISMA-P Group. Preferred reporting items for systematic review and meta-analysis protocols (PRISMA-P) 2015: elaboration and explanation. BMJ. 2015 Jan 2;349(jan02 1):g7647.

For peer review only

BMJ Open

Interventions to improve perinatal outcomes amongst migrant women in high-income countries: a systematic review protocol

Journal:	*BMJ Open*
Manuscript ID	bmjopen-2023-072090.R2
Article Type:	Protocol
Date Submitted by the Author:	28-Jul-2023
Complete List of Authors:	Stevenson, Kerrie; University College London, Institute of Health Informatics<br>Ogunlana, K; University College London, Institute of Health Informatics<br>Edwards, Samuel; University of Warwick, Warwick Medical School<br>Henderson, William; University College London, Library Services<br>Rayment-Jones, Hannah; King's College London, Women and Children's Health<br>McGranahan, Majel; University of Warwick<br>Marti-Castaner, Maria; University of Copenhagen, Health Department of Public Health<br>Fellmeth, Gracia; University of Oxford Nuffield Department of Population Health<br>Luchenski, Serena; University College London, Institute of Health Informatics<br>Stevenson, Fiona; University College London, Primary Care and Population Sciences<br>Knight, Marian; University of Oxford, National Perinatal Epidemiology Unit<br>Aldridge, Robert; University College London, Institute of Health Informatics
**Primary Subject Heading**:	Obstetrics and gynaecology
Secondary Subject Heading:	Public health
Keywords:	OBSTETRICS, PUBLIC HEALTH, PAEDIATRICS

SCHOLARONE™
Manuscripts

Interventions to improve perinatal outcomes amongst migrant women in high-income countries: a systematic review protocol

Authors

K Stevenson[1*], K Ogunlana[1], S Edwards[2], W Henderson[3], H Rayment-Jones[4], M McGranahan[2], M Marti-Castaner[5], G Fellmeth[6], S Luchenski[7], F Stevenson[8], M Knight[5], R Aldridge[1]

Affiliations

1. Institute of Health Informatics, University College London, 222 Euston Road, London NW1 2DA, UK.
2. Warwick Medical School, University of Warwick, Coventry CV4 7HL, UK.
3. Library Services, University College London, Gower Street, London WC1E 6BT, UK.
4. Department of Women and Children's Health, King's College London, Strand, London WC2R 2LS, UK.
5. Danish Research Centre for Migration, Ethnicity, and Health, Department of Public Health, University of Copenhagen, Øster Farimagsgade 5,1014 København K, Denmark.
6. National Perinatal Epidemiology Unit, Nuffield Department of Population Health, University of Oxford, Old Road Campus, Headington, Oxford OX3 7LF, UK.
7. Collaborative Centre for Inclusion Health, University College London, 1-19 Torrington Place, London WC1E 7HB UK.
8. Research Department of Primary Care and Health, Royal Free Campus, Rowland Hill Street, London, NW33 2PF, UK.

***Correspondence to:**

Dr Kerrie Stevenson

Institute of Health Informatics, University College London, 222 Euston Road, London NW1 2DA, UK

k.stevenson@ucl.ac.uk

ABSTRACT

Introduction

For peer review only

Women who are migrants and who are pregnant or postpartum are at high risk of poorer perinatal outcomes compared to host country populations due to experiencing numerous additional stressors including social exclusion and language barriers. High-income countries (HICs) host many migrants, including forced migrants who may face additional challenges in the peripartum period. Although HICs' maternity care systems are often well developed, they are not routinely tailored to the needs of migrant women. The primary objective will be to determine what interventions exist to improve perinatal outcomes for migrant women in HICs. The secondary objective will be to explore the effectiveness of these interventions by exploring the impact on perinatal outcomes. The main outcomes of interest will be rates of pre-term birth, birthweight, and number of antenatal or postnatal appointments attended.

Methods and analysis

This protocol follows the Preferred Reporting Items for Systematic Reviews and Meta-Analyses Protocols guidelines. EMBASE, EMCARE, MEDLINE and PsycInfo, CENTRAL, Scopus, CINAHL Plus, and Web of Science, as well as grey literature sources will be searched from inception up to December 2022. We will include randomised-controlled trials, quasi-experimental and interventional studies of interventions which aim to improve perinatal outcomes in any HIC. There will be no language restrictions. We will exclude studies presenting only qualitative outcomes and those including mixed populations of migrant and non-migrant women. Screening and data extraction will be completed by two independent reviewers and risk of bias will be assessed using the Quality Assessment Tool for Quantitative Studies. If a collection of suitably comparable outcomes is retrieved, we will perform meta-analysis applying a random effects model. Presentation of results will comply with guidelines in the Cochrane Handbook of Systematic Reviews of Interventions and the PRISMA statement.

Ethics and dissemination

Ethical approval is not required. Results will be submitted for peer-reviewed publication and presented at national and international conferences. The findings will inform the work of the Lancet Migration European Hub.

For peer review only

PROSPERO registration number

CRD42022380678.

Strengths and limitations of this study

- We will employ rigorous methodology in accordance with the Cochrane Handbook of Systematic Reviews and report in accordance with the Preferred Reporting Items for Systematic Reviews and Meta-Analyses Protocol (PRISMA-P) statement.
- We aim to assess if included studies employed co-production methods at any point in intervention development or analysis.
- The search strategy was developed with an experienced medical librarian and adapted for each database searched.
- No language restrictions will be employed, and we are doing an extensive and systematic grey literature search which is often omitted in similar reviews.
- The certainty of evidence may be limited by the number of studies available and because some studies may be of low quality without a quantitative outcome assessment.

For peer review only

INTRODUCTION

Rationale

There are an estimated 281 million international migrants in the world, constituting 3.6% of the global population.[1] Of these, approximately 13% are forced migrants, including refugees and asylum seekers who often face significant hardships during migration and when settled in their host country.[2] The remainder are economic migrants who often choose to migrate to reunite with family or for better job prospects, but they may also experience marginalisation due to poverty, social isolation, and discrimination.[1] Between 2000 and 2022 the international migrant population increased by 108 million, and although the majority of international migrants originate from low- and middle-income countries (LMICs), increasing numbers hope

to settle in high-income countries (HICs).[1] HICs are not always well adapted to care for the needs of marginalised migrants, and many HICs struggle to adapt to this changing migration landscape.[1]

Women constitute approximately half of the international migrant population.[2] Women who migrate and who are pregnant or post-partum can face significant barriers to accessing maternity care, putting them at increased risk of poor mental and physical health.[3-6] They may experience poor social support in their host country, an inability to access to healthcare, and language barriers.[5, 7] Forced migrant women who are pregnant or post-partum experience additional challenges including pre-migration stressors such as the trauma of war, transition stressors including dangerous migration journeys and gender-based violence, and post-migration stressors such as poor access to legal entitlements, discrimination, and socio-cultural barriers in obstetric care.[7, 8] They are also more likely to have poor access to antenatal care and experience higher rates of perinatal mortality, miscarriage, and stillbirth than non-refugee women.[9] HICs often have some of the resources needed to support marginalised pregnant and postpartum migrant women, but often struggle to provide optimum care.[8]

To our knowledge no previous review has sought to quantitatively synthesise the literature on the most effective interventions to improve perinatal outcomes for migrant women in HICs. Balaam et al. conducted a systematic review in 2020 which aimed to identify social support interventions for asylum-seeking and refugee women in Europe.[10] The findings were qualitatively synthesised and women valued peer support and interventions that addressed their needs in a holistic way.[10] The UK National Institute of Health and Care Research (NIHR) commissioned a systematic review to explore interventions to improve maternity care for migrant women in the UK in 2017.[11] The review included only UK studies and identified just four interventions. These included peer support and specialist maternity care

For peer review only

interventions, but they were not robustly evaluated so it was difficult to draw conclusions on their effectiveness.[11]

Objectives

This systematic review aims to identify the most effective interventions to improve perinatal outcomes for migrant women in HICs by quantitatively synthesising the literature. The primary objective will be to determine what interventions exist to improve perinatal outcomes for migrant women in HICs. The secondary objective will be to explore the effectiveness of these interventions by exploring the impact on perinatal outcomes. The main outcomes of interest will be rates of pre-term birth, birthweight, and number of antenatal or postnatal appointments attended.

METHODS AND ANALYSIS

Registration and protocol adherence

This systematic review will be reported in accordance with the Preferred Reporting Items for Systematic Reviews and Meta-Analyses (PRISMA) guidelines and was registered on PROSPERO: CRD42022380678 on 9th December 2022.

Definitions

For the purposes of this review, migrant women will be defined as being aged 16 years or older and who were born outside their host country. Eligible maternity care interventions are any hospital- or community-based initiatives offered in the antenatal, perinatal, or postnatal period up to one year postpartum. The perinatal period is defined as pregnancy and up to one year postpartum.

Eligibility criteria and patient, intervention, comparison, and outcome (PICO) framework

PICO framework

For peer review only

- Population: Perinatal migrant women (those who were not born in their host country) aged 16 years or older and living in HICs (defined as being in the World Bank high-income economy category).[12]
- Intervention: Any hospital- or community-based activity undertaken with the aim of improving perinatal outcomes and delivered during the antenatal period and up to one year postpartum.
- Control: Usual care if data are available.
- Outcome: The main outcomes of interest will be rates of pre-term birth, birthweight, and number of antenatal or postnatal appointments attended as these are crucial measures of quality of maternity care in accordance with the World Health Organisation guidelines for Quality of Care for Pregnant Women and Newborns.[13] Improvements in perinatal outcomes (rates of miscarriage, preterm birth, stillbirth, birthweight, mode of delivery, APGAR score, maternal/neonatal critical care admission, breastfeeding initiation and duration, maternal/neonatal death, perinatal mental illness); number of antenatal or postnatal appointments attended; or change in maternal wellbeing as assessed by validated mental illness or wellbeing screening scales, as well as any other outcomes retrieved from included studies.

Inclusion and exclusion criteria

Observational, quasi-experimental, and experimental intervention studies published from inception will be included. Abstracts, non-empirical research, opinion, or editorial pieces will be excluded. If duplicate reports or publications of the same data are retrieved, the less complete or recent version will be excluded if the same data are reported. Studies including only a qualitative outcome assessment will be excluded. Interventions that were not specifically designed or adapted for migrant women in the perinatal period will be excluded. This is to ensure our results are focused on interventions that could be directly implemented for migrant women and have direct impact their outcomes. Additionally, we felt this ensures the systematic review is focused and won’t return an unmanageable number of results.

For peer review only

Patient and public involvement

Migrant women who are residing in the UK helped with the review's inception and design, and will also help with data extraction, analysis, and interpretation. They will also help with disseminating the work through co-authorship on peer-reviewed manuscripts and presentations at conferences.

Search strategy and data sources

EMBASE, EMCARE, MEDLINE and PsycInfo via Ovid, Cochrane Central Register of Controlled Trials (CENTRAL) via Cochrane Library, Scopus, CINAHL Plus via EBSCOHost, and Web of Science from inception up until December 2022 (Appendix 1). Grey literature sources including Google Scholar and trial registries were searched up until December 2022. The first 150 results from the following supplementary sources will be searched: Google Scholar, WHO International Clinical Trials Registry Platform (ICTRP), Clinicaltrials.gov, the World Health Organisation Website, and the UN Refugee Agency website. Forward citation searching will be employed on all included articles, and the reference lists of all included articles will also be searched. No language restrictions will be employed. If studies are published in a language other than English, one of the research team who is fluent in that language will assess for likely relevance and extract the data, if appropriate. The article will also be translated using Google Translate™ and a second reviewer will review its relevance and extract the data, if appropriate. If none of the research team are fluent in the language, we will pay UCL's graduate linguistics programme to translate article. The Boolean operators "AND" and "OR" will be employed to combine the descriptors. An experienced medical librarian helped to develop the search strategy and it has been adapted for each database. A search strategy is provided in Appendix 1. EndNote™ will be used to collect and manage studies retrieved.[14] Covidence™ will be used for deduplication and for study selection.[15]

For peer review only

Data extraction (selection and coding)

Two independent reviewers will screen the titles and abstracts of all the citations for relevance. Full text manuscripts for relevant articles will be obtained. Full texts will be independently assessed for eligibility using a checklist of the inclusion and exclusion criteria by two independent reviewers. Studies meeting the inclusion criteria will be selected for inclusion in the review. All excluded articles from the full text screening will be retained with reason for exclusion noted. Disagreements between reviewers will be discussed and agreement sought from a third reviewer if necessary. If data are not accessible from the paper, the authors will be contacted. All extracted data will be recorded on the piloted data extraction form by two separate reviewers and cross-checked. The main data fields will be: (a) Author, publication year; (b) Country; (c) Study design; (d) Population and baseline characteristics; (e) Context (community or hospital-based or online); (f) Intervention details; (g) Control or comparison; (h) Timing of outcome measurements; (i) Outcome measures (type, scale used, scale validation status); (j) Outcomes; (k) Quality assessment; (l) Reported according to CONSORT guidelines; (m) Co-production methods used. A flowchart will summarise the selection process in line with the PRISMA 2020 guidelines. Study characteristics will be summarised and presented in tables.

Quality assessment and risk of bias

Two reviewers will perform the critical appraisal independently and this will be independently cross-checked. The Quality Assessment Tool for Quantitative Studies will be used to assess rigor for each included study.[16] This was chosen as it has been developed and validated to assess both observational and experimental studies and shows reliability and validity. It assesses selection bias, study design, confounders, blinding, data collection, withdrawals, intervention integrity, and statistical analysis. To assess time-related biases we will include components of the Risk of Bias in Non-Randomised Studies (ROBINS-I) tool.[17] If meta-analysis is conducted, publication bias will be assessed using a funnel plot.

For peer review only

Data synthesis

Study characteristics including outcomes e.g., raw proportions, mean scores, and odds/risk ratios will be extracted and presented in tabular form. Narrative synthesis will be conducted according to Cochrane guidance and will include the creation of categories of interventions based on included papers e.g. specialist midwifery services, interpreting services, and use of a doula.[18, 19] If appropriate, heterogeneity between studies will be explored using the I^2 statistic. Random effects meta-analysis will be conducted with 95% confidence intervals to allow for expected heterogeneity between different study populations, if appropriate. If possible, pooled estimates of odds/risk ratios with 95% confidence intervals will be calculated to explore outcomes among migrant women compared to usual care. Subgroup analyses according to migration status (economic and forced) and study context (hospital- or community-based) will be conducted, if appropriate. Sensitivity analysis according to study quality and method of recruitment will be undertaken. If meta-analysis is not possible due to a lack of standardised outcome data, alternative quantitative methods will be used following the Cochrane Handbook for Systematic Reviews of Interventions guidance.[19] The handbook details several approaches, but it is likely a Harvest Plot will be most appropriate. A Harvest Plot provides a visual extension of vote counting by categorising studies based on their effect (e.g. ‘beneficial effect’ or ‘detrimental effect’).[20] Vote counting is recommended when there are inconsistent effect measures across studies. Traditional vote counting methods using statistical significance, magnitude of effect, or subjective rules have been shown to be misleading.[19] Instead, we will create a standardised binary metric, ‘beneficial’ or ‘detrimental’ based on direction of effect for each intervention according to outcome category (e.g. birthweight, stillbirth rates, or antenatal appointment attendance).[19] We will calculate the proportion of beneficial studies, 95% confidence interval (binomial exact calculation), and p-value (binomial probability test) to demonstrate if there is any evidence of an effect. We will present the findings on a Harvest Plot which will take the form of a ‘supermatrix’ which visually displays the vote counting results for each intervention.[19] All

For peer review only

statistical analyses will be conducted using R Studio.[21] Meta-biases such as publication bias across studies will not be assessed.

Confidence in cumulative evidence

Confidence in the strength of evidence found will be assessed using the overall assessment outlined in the Quality Assessment Tool for Quantitative Studies checklist.[16] This will be done by two reviewers and possible disagreement will be assessed by third reviewer.

ETHICS AND DISSEMINATION

Ethical approval is not required for this study as no primary data are being collected. We intend to publish the results in a peer-reviewed open access publication and present results at national and international conferences. The findings will inform the work of the Lancet Migration European Regional Hub.

DISCUSSION

This systematic review will provide a comprehensive overview of interventions being used to improve perinatal outcomes for migrant women in HICs. It will make a systematic assessment of the most effective interventions which will help to inform policy and clinical decision-making across these regions. The findings can also be used by researchers planning or adapting maternity care interventions for migrant women in HICs, as well as to guide resource allocation decisions by funders and healthcare managers. Potential limitations include the retrieval of low- quality studies with poor evaluation of outcomes which will inhibit our ability to robustly assess the evidence of effectiveness of these interventions. Attempts to retrieve all available literature have been utilised such as involving a medical librarian in the development of our search strategy to ensure a broad and sensitive selection of search terms and searching multiple databases and additional supplementary and grey

For peer review only

literature sources, however it is possible that some relevant studies/data may be missed. The certainty of evidence may also be limited by few studies including quantitative outcome assessments of interventions. Additionally, we are only including studies conducted in HICs which means we may miss effective interventions which were developed and tested in LMICs. Studies including antenatal interventions and subsequent perinatal outcomes can be affected by time-related biases if women are recruited at various points throughout their pregnancy, i.e. person-time of observation is not properly accounted for in the design or analysis of a study.[22] We are unable to adjust for these biases in quantitatively synthesising our findings, but will consider the implications of this in our conclusions. We are taking a co-production approach to the planning, conducting, and interpretation of this work by ensuring migrant women who have given birth or been pregnant in their host country are involved throughout. We will also be assessing if included studies have taken a co-production approach at any point in intervention development, implementation, or evaluation to gain an understanding of the prevalence of co-production in this field. We will identify gaps in the evidence to guide future research priorities.

Contributors

KS, KO, FS, MN, RA conceived of the review. KS, KO, SE, WH, HRJ, MM, MMC, GF, SL, FS, MN, RA developed the approach and protocol. KS developed the search strategy. KS wrote the first draft of the protocol, and all authors were invited to comment. KS is the guarantor of the review. All authors approved the final version of the protocol.

Funding

KS is supported by a National Institute of Health and Care Research (NIHR) Doctoral Fellowship [Grant number NIHR302577]. GF is funded by a Clinical Research Fellowship from the Nuffield Department of Population Health, University of Oxford [Grant number N/A]. MM is supported by a Medical Research Council Clinical Research Training Fellowship

For peer review only

[Grant number MR/W01498X/1]. The funders played no part in the design and writing of the protocol.

Competing interests

None declared.

References

1. UN Migration, *World Migration Report 2022*. 2022.
2. UN Refugee Agency. *Figures at a glance*. 2022; Available from: https://www.unhcr.org/uk/figures-at-a-glance.html.
3. Sudbury, H. and A. Robinson, *Barriers to sexual and reproductive health care for refugee and asylum-seeking women.* British Journal of Midwifery, 2016. **24**(4): p. 275-281.
4. Fair, F., et al., *Migrant women's experiences of pregnancy, childbirth and maternity care in European countries: a systematic review.* PLoS ONE, 2020. **15**(2).
5. Almeida, L.M., et al., *Maternal healthcare in migrants: A systematic review.* Maternal and Child Health Journal, 2013. **17**(8): p. 1346-1354.
6. Anderson, F.M., et al., *Prevalence and risk of mental disorders in the perinatal period among migrant women: a systematic review and meta-analysis.* Arch Womens Ment Health, 2017. **20**(3): p. 449-62.
7. Fair, F., et al., *Migrant women's experiences of pregnancy, childbirth and maternity care in European countries: A systematic review.* PLoS ONE, 2020. **15**(2): p. e0228378.
8. World Health Organization Regional Office for Europe, *Improving the health care of pregnant refugee and migrant women and newborn children*. 2018.
9. Sturrock, S., E. Williams, and A. Greenough, *Antenatal and perinatal outcomes of refugees in high income countries.* Journal of perinatal medicine, 2020. **49**(1): p. 80-93.
10. Balaam, M.-C., C. Kingdon, and M. Haith-Cooper, *A Systematic Review of Perinatal Social Support Interventions for Asylum-seeking and Refugee Women Residing in Europe.* Journal of Immigrant and Minority Health, 2021. **24**(3): p. 741-758.
11. Higginbottom, G.M.A., et al., *Experience of and access to maternity care in the UK by immigrant women: A narrative synthesis systematic review.* BMJ Open, 2019. **9**(12): p. e029478.
12. The World Bank. *World Bank Country and Lending Groups*. 2022; Available from: https://datahelpdesk.worldbank.org/knowledgebase/articles/906519.
13. Tunçalp, Ö., et al., *Quality of care for pregnant women and newborns-the WHO vision.* BJOG : an international journal of obstetrics and gynaecology, 2015. **122**(8).
14. Endnote, *EndNote*. 2013, Clarivate: Philadelphia, PA.

For peer review only

15. Veritas, *Covidence Systematic Review Software*. 2021, Veritas Health Innovation: Melbourne, Australia.
16. Effective Public Healthcare Panacea Project. *Quality Assessment Tool for Quantitative Studies*. 2022; Available from: https://www.ephpp.ca/quality-assessment-tool-for-quantitative-studies/.
17. Sterne, J., et al., *ROBINS-I: a tool for assessing risk of bias in non-randomised studies of interventions.* BMJ (Clinical research ed.), 2016. **355**.
18. Popay, J., et al., *Guidance on the Conduct of Narrative Synthesis in Systematic Reviews*. 2006.
19. Cochrane Collaboration Training, *Cochrane Handbook for Systematic Reviews of Interventions*. 2022.
20. Ogilvie, D., et al., *The harvest plot: a method for synthesising evidence about the differential effects of interventions.* BMC medical research methodology, 2008. **8**.
21. RStudio Team, *RStudio*, in *R: A language and environment for statistical computing*. 2020: Vienna, Austria.
22. Ukah, U., et al., *Time-related biases in perinatal pharmacoepidemiology: A systematic review of observational studies.* Pharmacoepidemiology and drug safety, 2022. **31**(12).

For peer review only

APPENDIX 1 – Search Strategies

Emcare

1 (Aruba* or Andorra* or "United Arab Emirates" or UAE or Emirati* or Antigua* or Barbuda* or Australia* or Austria* or Belgi* or Bahrain* or Baham* or Bermuda* or Barbad* or Brunei* or Canada* or Switzerland or Swiss or "Channel Island*" or Chile or Chilean* or Curacao* or "Cayman Island*" or Caymanian* or Cypr* or Czech* or German* or Denmark or Danish or Spain* or Estonia* or Finland or Finnish or France or French or Faroe* or "United Kingdom" or UK or England or Wales or welsh or Scotland or Brit* or Scot* or English or Gibraltar * or Gree* or Greenland* or Guam* or Chamorros or Hong Kong* or Croatia* or Hungar* or "Isle of Man" or Manx or Ireland or Irish or Iceland* or Israel* or Ital* or Japan* or "St Kitts" or Korea* or Kuwait* or Liechtenstein* or Lithuania* or Luxembourg* or Latvia* or Maca* or "St Martin*" or "Sint Maarten*" or Monaco or Monegasqu* or Malta or Maltese or "Northern Mariana Island*" or "New Caledonia*" or Netherlands or Dutch or Norw* or Nauru* or "New Zealand" or Kiwi or Oman* or Palau* or Poland or Polish or "Puerto Ric*" or Portug* or "French Polynesia*" or Qatar* or "Saudi Arabia*" or Singapor* or "San Marino" or "Sammarinese" or Slovak* or Slovenia* or Sweden or Swedish or Seychell* or Creole or "Turks Caicos" or Trinidad* or Tobago* or Uruguay* or "United States" or USA or America* or Venezuela* or "Virgin Island*" or EU or "European Union" or European*).ti,ab. 1458038

2 developed country/ or european union/ 13481

3 developed countr*.mp. 13352

4 (high income countr* or high-income countr*).mp. 6622

5 industrialised countr*.mp. 623

6 more economically developed countr*.mp. 22

7 MEDC.mp. 8

8 advanced countr*.mp. 158

9 advanced econom*.mp. 92

10 developed econom*.mp. 161

11 industrialized econom*.mp. 11

12 industrialised econom*.mp. 8

13 (Migrant* or refugee* or immigrant* or foreigner* or newcomer* or new-comer* or migration or immigration or emigrant* or nonnative* or non-native* or diaspora or border crossing* or transient* or asylum-seek*).mp. 163314

14 ((displaced or undocumented or foreign* or resettle* or settle* or displace*) adj3 (person* or people)).mp. 1301

15 exp Migration/ 8976

16 exp Refugee/ 7217

17 13 or 14 or 15 or 16 163953

For peer review only

18 (pregnan* or postpartum or post-partum or postnatal or post-natal or puerper* or antenatal or ante-natal or prenatal or pre-natal or antepartum or ante-partum or peripartum or peri-partum or birth* or trimester* or mother* or mum or mums or mom or moms or perinatal or peri-natal or childbirth* or obstetric* or maternity or maternal).mp. 460639

19 exp perinatal period/ or exp puerperium/ or exp pregnancy/ 125861

20 18 or 19 464076

21 (intervention* or prevent* or program* or strateg* or class* or special*).mp. 2320749

22 (Peer-led or "peer led" or doula* or midwi*).mp. 22932

23 ((antenatal or Postnatal) and (session* or lesson* or workshop*)).mp. 1060

24 21 or 22 or 23 2333617

25 1 and 17 and 20 and 24 2274

26 2 or 3 or 4 or 5 or 6 or 7 or 8 or 9 or 10 or 11 or 12 27873

27 17 and 20 and 26 212

28 25 or 27 2410

Embase

1 (Aruba* or Andorra* or "United Arab Emirates" or UAE or Emirati* or Antigua* or Barbuda* or Australia* or Austria* or Belgi* or Bahrain* or Baham* or Bermuda* or Barbad* or Brunei* or Canada* or Switzerland or Swiss or "Channel Island*" or Chile or Chilean* or Curacao* or "Cayman Island*" or Caymanian* or Cypr* or Czech* or German* or Denmark or Danish or Spain* or Estonia* or Finland or Finnish or France or French or Faroe* or "United Kingdom" or UK or England or Wales or welsh or Scotland or Brit* or Scot* or English or Gibraltar * or Gree* or Greenland* or Guam* or Chamorros or Hong Kong* or Croatia* or Hungar* or "Isle of Man" or Manx or Ireland or Irish or Iceland* or Israel* or Ital* or Japan* or "St Kitts" or Korea* or Kuwait* or Liechtenstein* or Lithuania* or Luxembourg* or Latvia* or Maca* or "St Martin*" or "Sint Maarten*" or Monaco or Monegasqu* or Malta or Maltese or "Northern Mariana Island*" or "New Caledonia*" or Netherlands or Dutch or Norw* or Nauru* or "New Zealand" or Kiwi or Oman* or Palau* or Poland or Polish or "Puerto Ric*" or Portug* or "French Polynesia*" or Qatar* or "Saudi Arabia*" or Singapor* or "San Marino" or "Sammarinese" or Slovak* or Slovenia* or Sweden or Swedish or Seychell* or Creole or "Turks Caicos" or Trinidad* or Tobago* or Uruguay* or "United States" or USA or America* or Venezuela* or "Virgin Island*" or EU or "European Union" or European*).ti,ab. 5387298

2 developed country/ or european union/ 64842

3 developed countr*.mp. 65772

4 (high income countr* or high-income countr*).mp. 16400

5 industrialised countr*.mp. 1957

6 more economically developed countr*.mp. 49

For peer review only

7 MEDC.mp. 51

8 advanced countr*.mp. 668

9 advanced econom*.mp. 218

10 developed econom*.mp. 516

11 industrialized econom*.mp. 40

12 industrialised econom*.mp. 11

13 (Migrant* or refugee* or immigrant* or foreigner* or newcomer* or new-comer* or migration or immigration or emigrant* or nonnative* or non-native* or diaspora or border crossing* or transient* or asylum-seek*).mp. 1056344

14 ((displaced or undocumented or foreign* or resettle* or settle* or displace*) adj3 (person* or people)).mp. 2841

15 exp Migration/ 48713

16 exp Refugee/ 16436

17 13 or 14 or 15 or 16 1057673

18 (pregnan* or postpartum or post-partum or postnatal or post-natal or puerper* or antenatal or ante-natal or prenatal or pre-natal or antepartum or ante-partum or peripartum or peri-partum or birth* or trimester* or mother* or mum or mums or mom or moms or perinatal or peri-natal or childbirth* or obstetric* or maternity or maternal).mp. 2417880

19 exp perinatal period/ or exp puerperium/ or exp pregnancy/ 824664

20 18 or 19 2436673

21 (intervention* or prevent* or program* or strateg* or class* or special*).mp. 9841412

22 (Peer-led or "peer led" or doula* or midwi*).mp. 47440

23 ((antenatal or Postnatal) and (session* or lesson* or workshop*)).mp. 3201

24 21 or 22 or 23 9869943

25 1 and 17 and 20 and 24 6620

26 2 or 3 or 4 or 5 or 6 or 7 or 8 or 9 or 10 or 11 or 12 114481

27 17 and 20 and 26 956

28 25 or 27 7319

SCOPUS

TITLE-ABS-KEY (aruba* OR andorra* OR "United Arab Emirates" OR uae OR emirati* OR antigua* OR barbuda* OR australia* OR austria* OR belgi* OR bahrain* OR baham* OR bermuda* OR barbad* OR brunei* OR canada* OR switzerland OR swiss OR "Channel Islands" OR chile OR chilean* OR curacao* OR "Cayman Islands" OR caymanian* OR cypr* OR czech* OR german* OR denmark OR danish OR spain* OR estonia* OR finland OR finish OR france OR french OR faroe* OR

For peer review only

"United Kingdom" OR uk OR england OR wales OR scotland OR brit* OR scot* OR english OR "Gibraltar *" OR gree* OR greenland* OR guam* OR chamorros OR "Hong Kong*" OR croatia* OR hungar* OR "Isle of Man" OR manx OR ireland OR irish OR iceland* OR israel* OR ital* OR japan* OR "St Kitts" OR korea* OR kuwait* OR liechtenstein* OR lithuania* OR luxembourg* OR latvia* OR maca* OR "St Martin*" OR "Sint Maarten*" OR monaco OR monegasqu* OR malta OR maltese OR "Northern Mariana Island*" OR "New Caledonia*" OR netherlands OR dutch OR norw* OR nauru* OR "New Zealand" OR kiwi OR oman* OR palau* OR poland OR polish OR "Puerto Ric*" OR portug* OR "French Polynesia*" OR qatar* OR "Saudi Arabia*" OR singapor* OR "San Marino" OR sammarinese OR slovak* OR slovenia* OR sweden OR swedish OR seychell* OR creole OR "Turks Caicos" OR trinidad* OR tobago* OR uruguay* OR "United States" OR usa OR america* OR venezuela* OR "Virgin Island*" OR eu OR "European Union" OR european* OR "high income countr*" OR "high-income countr*" OR "industrialised countr*" OR "more economically developed countr*" OR "advanced countr*" OR "advanced econom*" OR "MEDC" OR "developed econom*" OR "industrialized econom*" OR "industrialised econom*") **AND** TITLE-ABS-KEY (migrant* OR refugee* OR immigrant* OR foreigner* OR newcomer* OR new-comer* OR migration OR immigration OR emigrant* OR nonnative* OR non-native* OR diaspora OR "border crossing*" OR transient* OR asylum-seek* OR "Displaced people*" OR "undocumented people*" OR "foreign* people*" OR "resettle* people*" OR "settle* people*" OR "Displaced person*" OR "undocumented person*" OR "foreign* person*" OR "resettle* person*" OR "settle* person*") **AND** TITLE-ABS-KEY (pregnan* OR postpartum OR post-partum OR postnatal OR post-natal OR puerperal OR antenatal OR ante-natal OR prenatal OR pre-natal OR antepartum OR ante-partum OR peripartum OR peri-partum OR birth* OR trimester* OR mother* OR mum OR mums OR mom OR moms OR perinatal OR peri-natal OR childbirth* OR obstetric* OR puerper* OR maternity OR maternal) **AND** TITLE-ABS-KEY ((intervention* OR prevent* OR program* OR strateg* OR class* OR special* OR peer-led OR "peer led" OR doula* OR midwi*) OR ((antenatal OR postnatal) AND (class* OR session* OR lesson* OR workshop*)))

8815

MEDLINE

Ovid MEDLINE(R) ALL <1946 to December 09, 2022>

1 (Aruba* or Andorra* or "United Arab Emirates" or UAE or Emirati* or Antigua* or Barbuda* or Australia* or Austria* or Belgi* or Bahrain* or Baham* or Bermuda* or Barbad* or Brunei* or Canada* or Switzerland or Swiss or "Channel Island*" or Chile or Chilean* or Curacao* or "Cayman Island*" or Caymanian* or Cypr* or Czech* or German* or Denmark or Danish or Spain* or Estonia* or Finland or Finnish or France or French or Faroe* or "United Kingdom" or UK or England or Wales or welsh or Scotland or Brit* or Scot* or English or Gibraltar * or Gree* or Greenland* or Guam* or Chamorros or Hong Kong* or Croatia* or Hungar* or "Isle of Man" or Manx or Ireland or Irish or Iceland* or Israel* or Ital* or Japan* or "St Kitts" or Korea* or Kuwait* or Liechtenstein* or Lithuania* or Luxembourg* or Latvia* or Maca* or "St Martin*" or "Sint Maarten*" or Monaco or Monegasqu* or Malta or Maltese or "Northern Mariana Island*" or "New Caledonia*" or Netherlands or Dutch or Norw* or Nauru* or "New Zealand" or Kiwi or Oman* or Palau* or Poland or Polish or "Puerto Ric*" or Portug*

For peer review only

or "French Polynesia*" or Qatar* or "Saudi Arabia*" or Singapor* or "San Marino" or "Sammarinese" or Slovak* or Slovenia* or Sweden or Swedish or Seychell* or Creole or "Turks Caicos" or Trinidad* or Tobago* or Uruguay* or "United States" or USA or America* or Venezuela* or "Virgin Island*" or EU or "European Union" or European*).ti,ab. 3264235

2 developed countries/ or european union/ 38606

3 developed countr*.mp. 59651

4 (high income countr* or high-income countr*).mp. 11176

5 industrialised countr*.mp. 1502

6 more economically developed countr*.mp. 36

7 MEDC.mp. 43

8 advanced countr*.mp. 492

9 advanced econom*.mp. 233

10 developed econom*.mp. 455

11 industrialized econom*.mp. 43

12 industrialised econom*.mp. 11

13 (Migrant* or refugee* or immigrant* or foreigner* or newcomer* or new-comer* or migration or immigration or emigrant* or nonnative* or non-native* or diaspora or border crossing* or transient* or asylum-seek*).mp. 782544

14 ((displaced or undocumented or foreign* or resettle* or settle* or displace*) adj3 (person* or people)).mp. 4461

15 exp Human Migration/ 27663

16 Refugees/ 12718

17 "Transients and Migrants"/ 13851

18 13 or 14 or 15 or 16 or 17 784919

19 (pregnan* or postpartum or post-partum or postnatal or post-natal or antenatal or ante-natal or prenatal or pre-natal or antepartum or ante-partum or peripartum or peri-partum or birth* or trimester* or mother* or mum or mums or mom or moms or perinatal or peri-natal or childbirth* or obstetric* or puerper* or maternity or maternal).mp. 1856432

20 exp peripartum period/ or exp postpartum period/ or exp pregnancy/ 1013266

21 19 or 20 1878022

22 (intervention* or prevent* or program* or strateg* or class* or special*).mp. 7862429

23 (Peer-led or "peer led" or doula* or midwi*).mp. 41688

24 ((antenatal or Postnatal) and (session* or lesson* or workshop*)).mp. 1994

25 22 or 23 or 24 7888583

26 1 and 18 and 21 and 25 4520

For peer review only

27 2 or 3 or 4 or 5 or 6 or 7 or 8 or 9 or 10 or 11 or 12 89366

28 18 and 21 and 27 1198

29 26 or 28 5360

Web of Science Search Strategy

Database: Web of Science Core Collection

Entitlements:

- WOS.IC: 1993 to 2022
- WOS.CCR: 1985 to 2022
- WOS.SCI: 1900 to 2022
- WOS.AHCI: 1975 to 2022
- WOS.BHCI: 2005 to 2022
- WOS.BSCI: 2005 to 2022
- WOS.ESCI: 2015 to 2022
- WOS.ISTP: 1990 to 2022
- WOS.SSCI: 1900 to 2022
- WOS.ISSHP: 1990 to 2022

Searches:

1: TS=(Aruba* OR Andorra* OR "United Arab Emirates" OR UAE OR Emirati* OR Antigua* OR Barbuda* OR Australia* OR Austria* OR Belgi* OR Bahrain* OR Baham* OR Bermuda* OR Barbad* OR Brunei* OR Canada* OR Switzerland OR Swiss OR "Channel Island*" OR Chile OR Chilean* OR Curacao* OR "Cayman Island*" OR Caymanian* OR Cypr* OR Czech* OR German* OR Denmark OR Danish OR Spain* OR Estonia* OR Finland OR Finnish OR France OR French OR Faroe* OR "United Kingdom" OR UK OR England OR Wales OR welsh OR Scotland OR Brit* OR Scot* OR English OR "Gibraltar *" OR Gree* OR Greenland* OR Guam* OR Chamorros OR "Hong Kong*" OR Croatia* OR Hungar* OR "Isle of Man" OR Manx OR Ireland OR Irish OR Iceland* OR Israel* OR Ital* OR Japan* OR "St Kitts" OR Korea* OR Kuwait* OR Liechtenstein* OR Lithuania* OR Luxembourg* OR Latvia* OR Maca* OR "St Martin*" OR "Sint Maarten*" OR Monaco OR Monegasqu* OR Malta OR Maltese OR "Northern Mariana Island*" OR "New Caledonia*" OR Netherlands OR Dutch OR Norw* OR Nauru* OR "New Zealand" OR Kiwi OR Oman* OR Palau* OR Poland OR Polish OR "Puerto Ric*" OR Portug* OR "French Polynesia*" OR Qatar* OR "Saudi Arabia*"

For peer review only

OR Singapor* OR "San Marino" OR Sammarinese OR Slovak* OR Slovenia* OR Sweden OR Swedish OR Seychell* OR Creole OR "Turks Caicos" OR Trinidad* OR Tobago* OR Uruguay* OR "United States" OR USA OR America* OR Venezuela* OR "Virgin Island*" OR EU OR "European Union" OR European* OR "high income countr*" OR "high-income countr*" OR "industrialised countr*" OR "more economically developed countr*" OR "advanced countr*" OR "advanced econom*" OR "MEDC" OR "developed econom*" OR "industrialized econom*" OR "industrialised econom*")

Date Run: Mon Dec 12 2022 13:10:27 GMT+0000 (Greenwich Mean Time)
Results: 10269737

2: TS=(migrant* OR refugee* OR immigrant* OR foreigner* OR newcomer* OR new-comer* OR migration OR immigration OR emigrant* OR nonnative* OR non-native* OR diaspora OR "border crossing*" OR transient* OR asylum-seek* OR "Displaced people*" OR "undocumented people*" OR "foreign* people*" OR "resettle* people*" OR "settle* people*" OR "Displaced person*" OR "undocumented person*" OR "foreign* person*" OR "resettle* person*" OR "settle* person*")

Date Run: Mon Dec 12 2022 13:12:33 GMT+0000 (Greenwich Mean Time)
Results: 1534587

3: TS=(pregnan* OR postpartum OR post-partum OR postnatal OR post-natal OR antenatal OR ante-natal OR prenatal OR pre-natal OR antepartum OR ante-partum OR peripartum OR peri-partum OR birth* OR trimester* OR mother* OR mum OR mums OR mom OR moms OR perinatal OR peri-natal OR childbirth* OR obstetric* OR puerper* OR maternity OR maternal)

Date Run: Mon Dec 12 2022 13:13:05 GMT+0000 (Greenwich Mean Time)
Results: 1676056

4: TS=((intervention* OR prevent* OR program* OR strateg* OR class* OR special* OR peer-led OR "peer led" OR doula* OR midwi*) OR ((antenatal OR postnatal) AND (class* OR session* OR lesson* OR workshop*)))

Date Run: Mon Dec 12 2022 13:13:46 GMT+0000 (Greenwich Mean Time)
Results: 12259143

5: #4 AND #3 AND #2 AND #1

Date Run: Mon Dec 12 2022 13:13:54 GMT+0000 (Greenwich Mean Time)
Results: 5662

For peer review only

APA PsycInfo <1806 to December Week 1 2022>

1 (Aruba* or Andorra* or "United Arab Emirates" or UAE or Emirati* or Antigua* or Barbuda* or Australia* or Austria* or Belgi* or Bahrain* or Baham* or Bermuda* or Barbad* or Brunei* or Canada* or Switzerland or Swiss or "Channel Island*" or Chile or Chilean* or Curacao* or "Cayman Island*" or Caymanian* or Cypr* or Czech* or German* or Denmark or Danish or Spain* or Estonia* or Finland or Finnish or France or French or Faroe* or "United Kingdom" or UK or England or Wales or welsh or Scotland or Brit* or Scot* or English or Gibraltar * or Gree* or Greenland* or Guam* or Chamorros or Hong Kong* or Croatia* or Hungar* or "Isle of Man" or Manx or Ireland or Irish or Iceland* or Israel* or Ital* or Japan* or "St Kitts" or Korea* or Kuwait* or Liechtenstein* or Lithuania* or Luxembourg* or Latvia* or Maca* or "St Martin*" or "Sint Maarten*" or Monaco or Monegasqu* or Malta or Maltese or "Northern Mariana Island*" or "New Caledonia*" or Netherlands or Dutch or Norw* or Nauru* or "New Zealand" or Kiwi or Oman* or Palau* or Poland or Polish or "Puerto Ric*" or Portug* or "French Polynesia*" or Qatar* or "Saudi Arabia*" or Singapor* or "San Marino" or "Sammarinese" or Slovak* or Slovenia* or Sweden or Swedish or Seychell* or Creole or "Turks Caicos" or Trinidad* or Tobago* or Uruguay* or "United States" or USA or America* or Venezuela* or "Virgin Island*" or EU or "European Union" or European*).ti,ab. 998036

2 developed countries/ 1444

3 developed countr*.mp. 6366

4 (high income countr* or high-income countr*).mp. 2276

5 industrialised countr*.mp. 162

6 more economically developed countr*.mp. 9

7 MEDC.mp. 4

8 advanced countr*.mp. 149

9 advanced econom*.mp. 164

10 developed econom*.mp. 377

11 industrialized econom*.mp. 54

12 industrialised econom*.mp. 3

13 (Migrant* or refugee* or immigrant* or foreigner* or newcomer* or new-comer* or migration or immigration or emigrant* or nonnative* or non-native* or diaspora or border crossing* or transient* or asylum-seek*).mp. 106941

14 ((displaced or undocumented or foreign* or resettle* or settle* or displace*) adj3 (person* or people)).mp. 1523

15 exp Human Migration/ 17211

16 13 or 14 or 15 108706

17 (pregnan* or postpartum or post-partum or postnatal or post-natal or antenatal or ante-natal or prenatal or pre-natal or antepartum or ante-partum or peripartum or peri-partum or birth* or trimester* or mother* or mum or mums or mom or moms or perinatal or peri-natal or childbirth* or obstetric* or puerper* or maternity or maternal).mp. 286207

For peer review only

18 exp Intrapartum Period/ or exp Antepartum Period/ or Perinatal Period/ or exp pregnancy/ or Postnatal Period/ or exp Birth/ or exp Prenatal Care/ 54584

19 17 or 18 287131

20 (intervention* or prevent* or program* or strateg* or class* or special*).mp. 1754113

21 (Peer-led or "peer led" or doula* or midwi*).mp. 5292

22 ((antenatal or Postnatal) and (session* or lesson* or workshop*)).mp. 857

23 20 or 21 or 22 1756811

24 1 and 16 and 19 and 23 1693

25 2 or 3 or 4 or 5 or 6 or 7 or 8 or 9 or 10 or 11 or 12 9141

26 16 and 19 and 25 69

27 24 or 26 1739

Cochrane Central Register of Controlled Trials

Issue 11 of 12, November 2022

Search Name: CL

Date Run: 22/12/2022 10:48:39

Comment:

ID	Search	Hits
#1	MeSH descriptor: [Developed Countries] explode all trees	52
#2	MeSH descriptor: [European Union] explode all trees	65
#3	(developed countr*):ti,ab,kw	4180
#4	(high income countr*):ti,ab,kw	2009
#5	(high-income countr*):ti,ab,kw	854
#6	(industrialised countr*):ti,ab,kw	459
#7	(economically developed countr*):ti,ab,kw	35
#8	(MEDC):ti,ab,kw	2
#9	(advanced countr*):ti,ab,kw	1500
#10	(advanced econom*):ti,ab,kw	957
#11	(developed econom*):ti,ab,kw	3125
#12	(industrialized econom*):ti,ab,kw	62

For peer review only

#13 (industrialised econom*):ti,ab,kw 62

#14 {OR #1-#13} 10948

#15 (Migrant*):ti,ab,kw 441

#16 (refugee):ti,ab,kw 444

#17 (immigrant*):ti,ab,kw 839

#18 (foreigner):ti,ab,kw 21

#19 (newcomer*):ti,ab,kw 48

#20 (new-comer*):ti,ab,kw 5

#21 (migration):ti,ab,kw 3759

#22 (immigration):ti,ab,kw 231

#23 (emigrant*):ti,ab,kw 238

#24 (nonnative*):ti,ab,kw 86

#25 (non-native*):ti,ab,kw 77

#26 (diaspora):ti,ab,kw 4

#27 (border crossing*):ti,ab,kw 16

#28 (transient*):ti,ab,kw 19243

#29 (asylum-seek*):ti,ab,kw 82

#30 ((displaced or undocumented or foreign* or resettle* or settle* or displace*) NEAR/3 (person* or people)):ti,ab,kw 89

#31 MeSH descriptor: [Human Migration] explode all trees 68

#32 MeSH descriptor: [Refugees] this term only 173

#33 MeSH descriptor: [Transients and Migrants] this term only 80

#34 {OR #15-#33} 24729

#35 (pregnan* or postpartum or post-partum or postnatal or post-natal or antenatal or ante-natal or prenatal or pre-natal or antepartum or ante-partum or peripartum or peri-partum or birth* or trimester* or mother* or mum or mums or mom or moms or perinatal or peri-natal or childbirth* or obstetric* or puerper* or maternity or maternal):ti,ab,kw 119742

#36 MeSH descriptor: [Peripartum Period] explode all trees 21

#37 MeSH descriptor: [Postpartum Period] explode all trees 1942

#38 MeSH descriptor: [Pregnancy] explode all trees 25029

#39 {OR #35-#38} 119890

#40 #14 AND #34 AND #39 54

For peer review only

Interface - EBSCOhost Research Databases
Search Screen - Advanced Search
Database - CINAHL Plus

#	Query	Limiters/Expanders	Results
S1	MH "developed countries"	Search modes - Boolean/Phrase	4,144
S2	(MH "European Union")	Search modes - Boolean/Phrase	6,324
S3	TX developed countr*	Search modes - Boolean/Phrase	14,015
S4	TX high income countr*	Search modes - Boolean/Phrase	5,938
S5	TX high-income countr*	Search modes - Boolean/Phrase	5,253
S6	TX industrialised countr*	Search modes - Boolean/Phrase	409
S7	TX economically developed countr*	Search modes - Boolean/Phrase	113
S8	TX MEDC	Search modes - Boolean/Phrase	9
S9	TX advanced countr*	Search modes - Boolean/Phrase	564
S10	TX advanced econom*	Search modes - Boolean/Phrase	406
S11	TX developed econom*	Search modes - Boolean/Phrase	1,039
S12	TX industrialized econom*	Search modes - Boolean/Phrase	50
S13	TX industrialised econom*	Search modes - Boolean/Phrase	11
S14	S1 OR S2 OR S3 OR S4 OR S5 OR S6 OR S7 OR S8 OR S9 OR S10 OR S11 OR S12 OR S13	Search modes - Boolean/Phrase	27,284
S15	TX Migrant* or refugee* or immigrant* or foreigner* or	Search modes - Boolean/Phrase	106,229

	newcomer* or new-comer* or migration or immigration or emigrant* or nonnative* or non-native* or diaspora or border crossing* or transient* or asylum-seek*		
S16	TX (displaced or undocumented or foreign* or resettle* or settle* or displace*) n3 (person* or people)	Search modes - Boolean/Phrase	1,998
S17	(MH "Refugees+") OR (MH "Transients and Migrants")	Search modes - Boolean/Phrase	14,390
S18	S15 OR S16 OR S17	Search modes - Boolean/Phrase	107,366
S19	TX pregnan* or postpartum or post-partum or postnatal or post-natal or antenatal or ante-natal or prenatal or pre-natal or antepartum or ante-partum or peripartum or peri-partum or birth* or trimester* or mother* or mum or mums or mom or moms or perinatal or peri-natal or childbirth* or obstetric* or puerper* or maternity or maternal	Search modes - Boolean/Phrase	502,585
S20	(MH "Pregnancy+")	Expanders - Apply equivalent subjects Search modes - Boolean/Phrase	246,130
S21	(MH "Postnatal Period+")	Expanders - Apply equivalent subjects Search modes - Boolean/Phrase	16,885

For peer review only

S22	(MH "Postnatal care+") OR (MH "Prenatal Care") OR (MH "Perinatal Care")	Search modes - Boolean/Phrase	29,500
S23	S19 OR S20 OR S21 OR S22	Search modes - Boolean/Phrase	504,522
S24	S14 AND S18 AND S23	Search modes - Boolean/Phrase	237
S25	TI (Aruba* or Andorra* or "United Arab Emirates" or UAE or Emirati* or Antigua* or Barbuda* or Australia* or Austria* or Belgi* or Bahrain* or Baham* or Bermuda* or Barbad* or Brunei* or Canada* or Switzerland or Swiss or "Channel Island*" or Chile or Chilean* or Curacao* or "Cayman Island*" or Caymanian* or Cypr* or Czech* or German* or Denmark or Danish or Spain* or Estonia* or Finland or Finnish or France or French or Faroe* or "United Kingdom" or UK or England or Wales or welsh or Scotland or Brit* or Scot* or English or Gibraltar * or Gree* or Greenland* or Guam* or Chamorros or Hong Kong* or Croatia* or Hungar* or "Isle of Man" or Manx or Ireland or Irish or Iceland* or Israel* or Ital* or Japan* or "St Kitts" or Korea* or Kuwait* or Liechtenstein* or	Search modes - Boolean/Phrase	1,194,855

For peer review only

	Lithuania* or Luxembourg* or Latvia* or Maca* or "St Martin*" or "Sint Maarten*" or Monaco or Monegasqu* or Malta or Maltese or "Northern Mariana Island*" or "New Caledonia*" or Netherlands or Dutch or Norw* or Nauru* or "New Zealand" or Kiwi or Oman* or Palau* or Poland or Polish or "Puerto Ric*" or Portug* or "French Polynesia*" or Qatar* or "Saudi Arabia*" or Singapor* or "San Marino" or "Sammarinese" or Slovak* or Slovenia* or Sweden or Swedish or Seychell* or Creole or "Turks Caicos" or Trinidad* or Tobago* or Uruguay* or "United States" or USA or America* or Venezuela* or "Virgin Island*" or EU or "European Union" or European*) OR AB (Aruba* or Andorra* or "United Arab Emirates" or UAE or Emirati* or Antigua* or Barbuda* or Australia* or Austria* or Belgi* or Bahrain* or Baham* or Bermuda* or Barbad* or Brunei* or Canada* or Switzerland or Swiss or "Channel Island*" or Chile or Chilean* or Curacao* or		

For peer review only

	"Cayman Island*" or Caymanian* or Cypr* or Czech* or German* or Denmark or Danish or Spain* or Estonia* or Finland or Finnish or France or French or Faroe* or "United Kingdom" or UK or England or Wales or welsh or Scotland or Brit* or Scot* or English or Gibraltar * or Gree* or Greenland* or Guam* or Chamorros or Hong Kong* or Croatia* or Hungar* or "Isle of Man" or Manx or Ireland or Irish or Iceland* or Israel* or Ital* or Japan* or "St Kitts" or Korea* or Kuwait* or Liechtenstein* or Lithuania* or Luxembourg* or Latvia* or Maca* or "St Martin*" or "Sint Maarten*" or Monaco or Monegasqu* or Malta or Maltese or "Northern Mariana Island*" or "New Caledonia*" or Netherlands or Dutch or Norw* or Nauru* or "New Zealand" or Kiwi or Oman* or Palau* or Poland or Polish or "Puerto Ric*" or Portug* or "French Polynesia*" or Qatar* or "Saudi Arabia*" or Singapor* or "San Marino" or "Sammarinese" or Slovak* or Slovenia* or Sweden or		

For peer review only

	Swedish or Seychell* or Creole or "Turks Caicos" or Trinidad* or Tobago* or Uruguay* or "United States" or USA or America* or Venezuela* or "Virgin Island*" or EU or "European Union" or European*)		
S26	S18 AND S23 AND S25	Search modes - Boolean/Phrase	4,466
S27	TX intervention* or prevent* or program* or strateg* or "classes" or "specialist" OR "specialists" OR Peer-led or "peer led" or doula* or midwi*	Search modes - Boolean/Phrase	2,112,926
S28	TX (antenatal or Postnatal) AND (session* or lesson* or workshop*)	Search modes - Boolean/Phrase	1,184
S29	S27 OR S28	Search modes - Boolean/Phrase	2,113,168
S30	S26 AND S29	Search modes - Boolean/Phrase	1,960
S31	S24 OR S30	Search modes - Boolean/Phrase	2,128

Grey Literature and Registry Searching:

WHO International Clinical Trials Registry Platform (ICTRP):

For peer review only

https://trialsearch.who.int/AdvSearch.aspx using Pregnancy AND migrant

Clinicaltrials.gov:

https://clinicaltrials.gov/ct2/search/advanced?cond=&term=&cntry=&state=&city=&dist= using the terms ‘Pregnancy’ ‘Migrant’ in the 'other terms' search field

Google Scholar:

Pregnancy refugee OR migrant OR asylum "high income"

World Health Organization Website:

site:who.int pregnancy refugee OR migrant OR asylum "high income": https://www.google.com/search?q=site%3Awho.int+pregnancy+refugee+OR+migrant+OR+asylum+%22high+income%22

UN Refugee Agency Website:

site:unhcr.org pregnancy refugee OR migrant OR asylum "high income": https://www.google.com/search?q=site%3Aunhcr.org+pregnancy+refugee+OR+migrant+OR+asylum+%22high+income%22

For peer review only

PRISMA-P (Preferred Reporting Items for Systematic review and Meta-Analysis Protocols) 2015 checklist: recommended items to address in a systematic review protocol*

Relevant Page number in 'red'.

Section and topic	Item No	Checklist item
**ADMINISTRATIVE INFORMATION**		
Title:		
Identification	1a	Identify the report as a protocol of a systematic review **Page 1**
Update	1b	If the protocol is for an update of a previous systematic review, identify as such **N/A**
Registration	2	If registered, provide the name of the registry (such as PROSPERO) and registration number **Page 3 & 5**
Authors:		
Contact	3a	Provide name, institutional affiliation, e-mail address of all protocol authors; provide physical mailing address of corresponding author **Page 1**
Contributions	3b	Describe contributions of protocol authors and identify the guarantor of the review **Page 12**
Amendments	4	If the protocol represents an amendment of a previously completed or published protocol, identify as such and list changes; otherwise, state plan for documenting important protocol amendments **N/A**
Support:		
Sources	5a	Indicate sources of financial or other support for the review **Page 12**
Sponsor	5b	Provide name for the review funder and/or sponsor **Page 12**
Role of sponsor or funder	5c	Describe roles of funder(s), sponsor(s), and/or institution(s), if any, in developing the protocol **Page 12**
**INTRODUCTION**		
Rationale	6	Describe the rationale for the review in the context of what is already known **Page 4**
Objectives	7	Provide an explicit statement of the question(s) the review will address with reference to participants, interventions, comparators, and outcomes (PICO) **Page 6**
**METHODS**		

For peer review only

Eligibility criteria	8	Specify the study characteristics (such as PICO, study design, setting, time frame) and report characteristics (such as years considered, language, publication status) to be used as criteria for eligibility for the review **Page 6 & 7**
Information sources	9	Describe all intended information sources (such as electronic databases, contact with study authors, trial registers or other grey literature sources) with planned dates of coverage **Page 7 & 8**
Search strategy	10	Present draft of search strategy to be used for at least one electronic database, including planned limits, such that it could be repeated **Appendix 1**
Study records: Data management	11a	Describe the mechanism(s) that will be used to manage records and data throughout the review **Page 7 & 8**
Selection process	11b	State the process that will be used for selecting studies (such as two independent reviewers) through each phase of the review (that is, screening, eligibility and inclusion in meta-analysis) **Page 7 & 8**
Data collection process	11c	Describe planned method of extracting data from reports (such as piloting forms, done independently, in duplicate), any processes for obtaining and confirming data from investigators **Page 7 & 8**
Data items	12	List and define all variables for which data will be sought (such as PICO items, funding sources), any pre-planned data assumptions and simplifications **Page 8**
Outcomes and prioritization	13	List and define all outcomes for which data will be sought, including prioritization of main and additional outcomes, with rationale **Page 6**
Risk of bias in individual studies	14	Describe anticipated methods for assessing risk of bias of individual studies, including whether this will be done at the outcome or study level, or both; state how this information will be used in data synthesis **Page 8 & 9**
Data synthesis	15a	Describe criteria under which study data will be quantitatively synthesised **Page 9**
	15b	If data are appropriate for quantitative synthesis, describe planned summary measures, methods of handling data and methods of combining data from studies, including any planned exploration of consistency (such as I^2, Kendall's τ) **Page 9 & 10**
	15c	Describe any proposed additional analyses (such as sensitivity or subgroup analyses, meta-regression) **Page 9 & 10**
	15d	If quantitative synthesis is not appropriate, describe the type of summary planned **Page 9 & 10**
Meta-bias(es)	16	Specify any planned assessment of meta-bias(es) (such as publication bias across studies, selective reporting within studies) **Page 10**
Confidence in cumulative evidence	17	Describe how the strength of the body of evidence will be assessed (such as GRADE) **Page 10**

*** It is strongly recommended that this checklist be read in conjunction with the PRISMA-P Explanation and Elaboration (cite when available) for important clarification on the items. Amendments to a review protocol should be tracked and dated.** **The copyright for PRISMA-P (including checklist) is held by the PRISMA-P Group and is distributed under a Creative Commons Attribution Licence 4.0.**

From: Shamseer L, Moher D, Clarke M, Ghersi D, Liberati A, Petticrew M, Shekelle P, Stewart L, PRISMA-P Group. Preferred reporting items for systematic review and meta-analysis protocols (PRISMA-P) 2015: elaboration and explanation. BMJ. 2015 Jan 2;349(jan02 1):g7647.

For peer review only
